# Application of Selection Mapping to Identify Genomic Regions Associated with Dairy Production in Sheep

**DOI:** 10.1371/journal.pone.0094623

**Published:** 2014-05-01

**Authors:** Beatriz Gutiérrez-Gil, Juan Jose Arranz, Ricardo Pong-Wong, Elsa García-Gámez, James Kijas, Pamela Wiener

**Affiliations:** 1 Dpto. Producción Animal, Universidad de León, León, Spain; 2 The Roslin Institute and R(D)SVS, University of Edinburgh, Roslin, Midlothian, United Kingdom; 3 Animal, Food and Health Sciences, CSIRO, Brisbane, Australia; Auburn University, United States of America

## Abstract

In Europe, especially in Mediterranean areas, the sheep has been traditionally exploited as a dual purpose species, with income from both meat and milk. Modernization of husbandry methods and the establishment of breeding schemes focused on milk production have led to the development of “dairy breeds.” This study investigated selective sweeps specifically related to dairy production in sheep by searching for regions commonly identified in different European dairy breeds. With this aim, genotypes from 44,545 SNP markers covering the sheep autosomes were analysed in both European dairy and non-dairy sheep breeds using two approaches: (i) identification of genomic regions showing extreme genetic differentiation between each dairy breed and a closely related non-dairy breed, and (ii) identification of regions with reduced variation (heterozygosity) in the dairy breeds using two methods. Regions detected in at least two breeds (breed pairs) by the two approaches (genetic differentiation and at least one of the heterozygosity-based analyses) were labeled as core candidate convergence regions and further investigated for candidate genes. Following this approach six regions were detected. For some of them, strong candidate genes have been proposed (e.g. *ABCG2, SPP1*), whereas some other genes designated as candidates based on their association with sheep and cattle dairy traits (e.g. *LALBA, DGAT1A*) were not associated with a detectable sweep signal. Few of the identified regions were coincident with QTL previously reported in sheep, although many of them corresponded to orthologous regions in cattle where QTL for dairy traits have been identified. Due to the limited number of QTL studies reported in sheep compared with cattle, the results illustrate the potential value of selection mapping to identify genomic regions associated with dairy traits in sheep.

## Introduction

Since their domestication 8 000–9 000 years ago (reviewed by [1]), sheep (*Ovis aries*) have been used by humans for the production of wool, meat and milk. Adaptation to very different geographic and climatic conditions and the specialization for specific characteristics have resulted in a phenotypically highly diverse species. The first documented modifications to sheep by human-imposed selection had taken place by the time that illustrations and records first appeared c. 3 000 BC and primarily concerned morphological and coat colour traits with the initial major morphological changes including reduction in the length of the legs, lengthening of the tail and alteration of horn shape [2]. Initially, sheep were kept solely for meat, milk and skins. Archaeological evidence suggests that selection for woolly sheep may have begun around 6000 BC.

Dairy sheep are mainly found in Europe, especially in Mediterranean areas, where they have been traditionally exploited as a dual purpose species, with income from both meat and milk. Sheep milk has a higher solid content than cow or goat milk, which means that it is particularly suited to processing into cheese. Historically, most sheep milk has been produced by multipurpose local breeds with low-to-medium milk yields and raised under traditional husbandry conditions [3]. More recently, modernization of husbandry methods and the establishment of breeding schemes focused on milk production have led to the development of “dairy breeds”, facilitated by the implementation of quantitative genetics-based breeding and the use of artificial insemination [2]. The market for sheep milk and sheep dairy products appears to be growing, even in those countries without a history of sheep dairying [4].

Selection sweep mapping strategies, in which regions of the genome are identified that show patterns consistent with positive selection, can be used as a complementary approach to linkage mapping and genome-wide association study (GWAS) analysis to identify regions of the genome that influence important traits in livestock. Various methods have been applied to livestock and other domesticated animals, with the aim of identifying genomic regions with characteristics that reflect the influence of selection: extended low diversity haplotypes [5], overall low heterozygosity (e.g. [6], [7]), specific diversity patterns [8], extreme allele frequencies [9] and between-breed differentiation [10], [11], [12]. Because of their well-documented selection pressures and highly-developed genetic resources, domesticated animal species also provide a valuable resource with the potential to identify the molecular pathways underlying phenotypic traits through the use of selection mapping approaches [10], [13].

To perform a search for signatures of selection related to dairy production in sheep, we used genotypes obtained with the *Illumina OvineSNP50 BeadChip* (Illumina Inc., San Diego, CA) for a number of European breeds genotyped within the framework of the Sheep HapMap Project [14]. These breeds include several selected primarily for dairy production and others not used for dairy. In order to specifically target regions under dairy-related selection and not related to other traits that may have been under selection in the sheep populations, only selection signatures commonly identified in different European dairy breeds were considered. We applied two approaches for the detection of selection sweeps: (i) we looked for regions with extreme genetic differentiation between each dairy breed and a closely related non-dairy breed, and (ii) we looked for regions of the genome with reduced heterozygosity in the dairy breeds using two methods. We then searched for candidate genes that could be selection targets within the regions that were identified in multiple breeds and using multiple analysis methods. For these regions we also looked for correspondence with previously reported QTL related to dairy production traits in cattle or sheep. Although the selection history of dairy cattle is quite different from that of dairy sheep, in particular because breeding schemes in sheep are focused on more localized (and in many cases isolated) breeds than the global dairy cattle population, comparison of our results with studies in cattle allowed us to evaluate whether some of the same regions/genes show evidence of selection in both dairy sheep and dairy cattle.

## Materials and Methods

### Data

#### Samples

We analysed a subset of the dataset generated in the Ovine HapMap project [14], which included 5 dairy and 5 non-dairy sheep breeds (Table 1).

**Table 1:** Breeds included in the present study.

Group	Breed name	Number ofsamples	Aptitude
Dairy	Chios	23	High milk production
	Churra	96	Double purpose breed(milk and lamb production
	Comisana	24	Highly-specialized dairy breed
	East Friesian Brown	39	Highly-specialized dairy breed
	Milk Lacaune	103	Highly-specialized dairy breed
Non-dairy	Australian Poll Merino	98	Meat production
	Meat Lacaune	78	Meat production
	Ojalada	24	Meat production
	Sakiz	22	Triple-purpose (milk, meat, wool)
	Finnsheep	99	Primary used for wool production;more recently used for meat production.

The classification established into Dairy and Non-dairy groups are presented together with some details about the breed aptitude.

#### Genotypes

After an initial quality control procedure described in detail elsewhere [14], this dataset provides the genotypes of 49,034 SNPs (using the *Illumina OvineSNP50 BeadChip*) distributed across the 26 autosomal ovine chromosomes and chromosome X (only one of the markers genotyped belongs to chromosome Y). Markers were filtered to exclude loci assigned to unmapped contigs. The analyses reported here focused on the remaining 44,545 of these SNP located on autosomes. The positions of the markers according to the Sheep Genome Assembly v2.0 (update September 2011) were used for the analyses.

### Selection Sweep Mapping Analysis Methods

- **Genetic differentiation: Pair-wise F_ST_ calculations.** In order to search for genomic regions that have been under divergent selection in dairy and non-dairy breeds, we examined genetic differentiation across the genome for five breed pairs. The selection of sheep breeds to serve as non-dairy partners for dairy breeds was based on the shortest divergence time estimates reported by the Sheep HapMap project (based on the extent of haplotype sharing and correlation of linkage disequilibrium values; Supplementary Information Figure S10 and Figure 3 in [14]), and close relationships according to additional Principal Component Analyses (PCA) performed in a selection of breeds (described in detail in File S1).

The following pairs of breeds of European ancestry were considered in the differentiation analysis:

  - Chios (Greek, dairy) *vs* Sakiz (Turkey, non-specialized)
  - Churra (Spanish, dairy) *vs* Ojalada (Spanish, meat)
  - Comisana (Italian, dairy) *vs* Australian Poll Merino (Australian, originated in southwest Europe, wool)
  - East Friesian Brown (highly specialized dairy) *vs* Finnsheep (Finland, primary wool, more recently used as a meat producing breed)
  - Milk Lacaune (French, highly specialized dairy) *vs* Australian Poll Merino (Australian, originated in southwest Europe, wool)
  - Milk Lacaune (French, highly specialized dairy) *vs* Meat Lacaune (French, meat)

For each of these pairs, unbiased estimates of Weir and Cockerham’s F_ST_ [15], a measure of genetic differentiation, were calculated as functions of variance components, as detailed in Akey et al. [16]. This type of approach to selection mapping, exploiting between-breed allele frequency differences, has been applied in studies of humans [16] and domesticated animals [10], [11], [12], [17], [18] where it has been demonstrated to be effective in identifying genes that are associated with breed differentiation.

- **Reduced diversity: Observed heterozygosity.** For all the breeds included in the pair-wise F_ST_ calculations, observed heterozygosity (ObsHtz) was calculated for each SNP marker. This approach has previously been applied in selection mapping studies of chickens [6], [7], pigs [19] and dogs [20].
- **Reduced diversity: Regression analysis for detection of regions with asymptotic heterozygosity patterns.** For all the breeds included in the pair-wise F_ST_ calculations, tests of significant asymptotic relationships between heterozygosity and distance from a test position were performed across the genome based on the approach of Wiener and Pong-Wong [8]. This method detects regions with patterns of variation consistent with positive selection: an asymptotic increase in marker variation (heterozygosity; *y*) with increasing distance (*x*) from a selected locus *y* = *A* +*B R* ^x^ (where R is the asymptotic rate of increase; B is the difference between heterozygosity at the test position and the asymptotic level; A is the asymptotic level of heterozygosity). For each regression (performed in Genstat, [21]), we recorded the parameters of the asymptotic regression, their standard errors, the significance level associated with the regression (p) and the variance explained by the curve. Positive and increasing regressions (0<R<1, B<0) were considered as being in the direction predicted by positive selection. Analysis of simulated data suggests improved precision of this selection mapping approach compared to an alternative haplotype-based method as well as robustness to demographic influences [8].

### Protocols for Selection Mapping Analyses

In order to determine appropriate parameters for the above-mentioned analyses, we investigated their behaviour on a test genomic region encompassing the myostatin (*GDF-8*) gene, which is known to have been under selection in the Texel breed (details in File S2).

#### Window/bracket sizes

Based on the analysis of the myostatin gene (File S2), window and bracket sizes for the three methods were established. For the differentiation and reduced heterozygosity analyses, F_ST_ and ObsHtz values, respectively, were averaged across sliding windows of 9 SNPs (F_ST_-9SNPW, ObsHtz-9SNPW). For the regression analysis, the test position was moved every 50 Kb across each chromosome and all markers within 10 Mb of this position (10 Mb-bracket size) were considered in the asymptotic regression. A –log(p) value was determined for each test position.

#### Identification of selection signals by individual methods

Evidence of positive selection was interpreted for window estimates in the extreme of the empirical distributions, as suggested by Akey et al. [10], [16] and employed in various subsequent studies (e.g. [11], [13]. Specifically, we considered the positions showing signatures of selection as the top 0.5th percent of the distributions for differentiation (F_ST_) and asymptotic regression (–log(p), for regressions in the predicted direction) or the bottom 0.5th percent for observed heterozygosity. Based on the results of the analysis of the myostatin gene (File S2), a selected “region” was defined as the range of positions within 2 Mb of each other showing evidence of selection by any of the three methods. An additional criterion for selected regions was that they were identified in at least two breed pairs, for F_ST_, or two dairy breeds, for heterozygosity-based methods (with distances up to 2 Mb allowed between the regions identified for different breeds). For genetic differentiation, we further required that regions of extreme F_ST_ must be detected in at least two different pairs of dairy – non-dairy breeds that did not share a common breed (e.g. top regions found only in the Milk Lacaune-Australian Poll Merino and Comisana-Australian Poll Merino but not in other studied pairs were not included in the list of differentiated regions). By requiring at least two breeds (or breed pairs) for the initial identification of candidate regions for each methodology, this selection mapping strategy will not identify dairy gene variants occurring in only one breed.

### Criteria for Identification of Regions with Shared Selection Signals

Based on the selected “regions” identified by the individual methods through the overlapping of at least two breeds or breed pairs, and taking into account that the F_ST_-based method is expected to specifically target traits relevant for dairy production, whereas signals detected by heterozygosity-based methods may not be specific for dairy-related selection, we defined a “convergent candidate region” (CCR) as one where a signal was identified by the pair-wise F_ST_ comparison and at least one of the reduced heterozygosity methods. Hence, a CCR was labelled where there was overlap between the position ranges of the candidate regions identified by the genetic differentiation methodology and at least one of the two heterozygosity-based methods, such that each CCR was associated with a region identified in at least two breeds (breed pairs) and using at least two different methods.

### Identification of Candidate Genes within CCR Regions

We identified the genes mapping to the end of each CCR using the genome browser of the sheep genome reference sequence (v2.0; http://www.livestockgenomics.csiro.au/cgi-bin/gbrowse/oarv2.0/) and identified the corresponding orthologous regions in the bovine genome (Cow (UMD3.1) using Ensembl (http://www.ensembl.org/Bos_taurus/Info/Index). A systematic extraction of all the annotated genes contained within the orthologous genomic ranges in cattle was performed using Biomart (www.biomart.org). Subsequently, an exhaustive search was performed for candidate genes previously linked to cattle dairy traits [22]. In addition, genes not included in this database but reported as candidate genes in the literature in relation to milk production or dairy-related traits were also identified. We also looked for correspondence with genes for which signatures of selection have been reported in studies of dairy cattle [23], [24], [25] and sheep [14], [26].

We evaluated correspondence of the CCR with QTL reported for milk production and other functional traits related to dairy production in sheep (based on the SheepQTL database, http://www.animalgenome.org/cgi-bin/QTLdb/OA/index). We also examined overlap between the CCR and QTL influencing milk-related traits, mastitis and other functional traits related to dairy production in cattle (based on the CattleQTL database; http://www.animalgenome.org/cgi-bin/QTLdb/BT/index), positioned on the bovine genome reference sequence (UMD_version 3.1).

## Results

### Regions Identified by Individual Methods

#### Genetic differentiation

**Figure 1:**
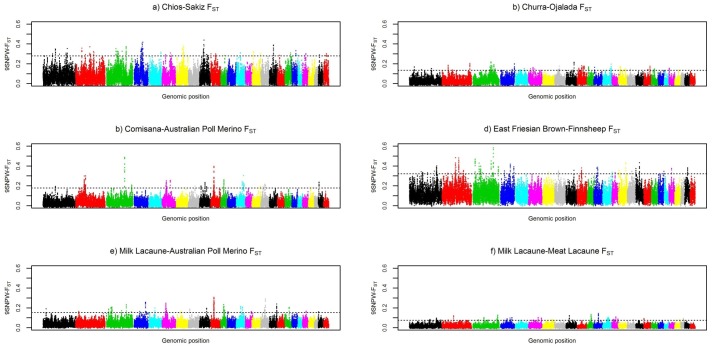
Genome-wide distribution of F_ST_ values for the six analysed breed pairs. The level of genetic differentiation, measured by F_ST_, was estimated within each dairy – non-dairy breed pair^1^, and averaged in sliding windows of 9 SNPs (F_ST_-9SNPW) across the genome: The horizontal line indicates the top 0.5.th percent threshold considered for the F_ST_-distributions. These raw results were used to identify F_ST_-based candidate regions (F_ST_-CRs) when overlapping significant selection signals (allowing gaps up to 2-Mb) were identified between different pairs. ^1^Breed pairs analysed: a) Chios-Sakiz, b) Churra-Ojalada; c) Comisana-Australian Poll Merino; d) East Friesian Brown -Finnsheep, e) Milk Lacaune-Australian Poll Merino f) Milk Lacaune-MeatLacune.

The level and range of the top 0.5% of F_ST_ values averaged in sliding windows of 9 SNPs (F_ST_-9SNPW) varied among the five breed pairs (Figure 1). The lowest genome-wide differentiation within a pair was found, as expected, for the Milk Lacaune-Meat Lacaune pair (0.076), whereas the highest levels of genetic differentiation were found for the East Friesian Brown-Finnsheep pair (0.752, for the 9SNP-window centered on marker OAR3_185527791) (Table 2).

**Table 2:** Maximum and minimum of the 0.005 top averaged pair-wise F_ST_ values in sliding windows of 9 SNPs (F_ST_-9SNPW) estimated for the pairs considered in the present work to detect selection signals in dairy sheep.

Breed pair	Min. F_ST_-9SNPW	Max. F_ST_ -9SNPW
Chios-Sakiz	0.2799	0.4392
Churra-Ojalada	0.1345	0.2193
Comisana-Australian Poll Merino	0.1781	0.4873
East Friesian Brown-Finnsheep	0.3212	0.7515
Milk Lacaune-Australian Poll Merino	0.1547	0.3071
Milk Lacaune-Meat Lacaune	0.0757	0.1449

Twenty-eight genomic regions distributed across 15 autosomes were identified in at least two dairy-non-dairy breed pairs (Table S1, where a reference number has been given to each of them: F_ST_-CandidateRegionX, F_ST_-CRX). The largest number of F_ST_-based candidate regions per chromosome was found on OAR3 (5 regions). The length of the F_ST_-based candidate regions varied from 0.215 Mb (OAR3, F_ST_-CR8) to 9.211 Mb (OAR6, F_ST_-CR14).

#### Reduced observed heterozygosity in dairy breeds

Fifty-five regions showing reduced observed heterozygosity (ObsHtz-CR1–ObsHtz-CR55) in more than one dairy breed were found across 21 of the 26 autosomes (Table S2; where a non-dairy breed showed reduced heterozygosity in the same region, this is also indicated). Eight of the candidate regions found in dairy breeds covered intervals larger than 3 Mb. The largest was that on OAR13 (ObsHtz-CR42; 56.061–63.781 Mb), followed by one on OAR6 (ObsHtz-CR27∶34.576–41.863 Mb), while the smallest region was a single window centered on marker on OAR2 (ObsHtz-CR9; 211.205 Mb). A normalized observed heterozygosity (NObsHtz) (based on that introduced by Rubin et al. [6]) was also calculated for all breeds analysed, again averaged in 9-SNP windows. There were no regions in the extreme lower end of the distribution (NObsHtz<-6) in the dairy breeds although the region on OAR6 (*ABCG2* gene region) had a value of −5.99 for the Meat Lacaune breed.

#### Regression analysis for detection of regions with asymptotic heterozygosity patterns in dairy breeds

Three regions ranging in size from 0.1 to 4.0 Mb were identified with asymptotic heterozygosity patterns (bracket size = 10 Mb) in two or more dairy breeds (RegBrack10-CR1–RegBrack10-CR3) (Table 3, where a non-dairy breed showed reduced heterozygosity in the same region, this is also indicated).

**Table 3:** Initial candidate regions identified on the basis of the regression analysis performed for detection of regions with asymptotic heterozygosity patterns in at least two of the dairy breeds (top 0.5% results for bracket sizes 5, 10 and 20 Mb).

Analysis	Regression-CR	Chr.	Dairybreed	Start position(Mb)	End position(Mb)	Non-dairybreed	Startposition (Mb)	Endposition (Mb)
Regression top0.5% bracket 5 Mb	RegBrack5-CR1	2	Churra	51.810	54.110	Ojalada	52.610	53.760
			Chios	52.860	53.410			
	RegBrack5-CR2	2	Milk Lacaune	104.360	104.560	Meat Lacaune	104.360	104.510
			Churra	104.460		Australian Poll Merino	104.410	104.460
	RegBrack5-CR3	2	Churra	122.360	122.910			
			Chios	123.010	123.210			
	RegBrack5-CR4	3	Milk Lacaune	75.192	75.292			
			Churra	75.292				
	RegBrack5-CR5	3	Milk Lacaune	168.742	168.892	Australian Poll Merino	168.692	168.942
			Churra	168.792	168.892	Meat Lacaune	168.792	168.892
	RegBrack5-CR6	6	Milk Lacaune	35.475	36.625	Meat Lacaune	34.725	36.775
			Comisana	36.625	37.325	Australian Poll Merino	35.975	37.175
	RegBrack5-CR7	11	Milk Lacaune	18.380	18.530	Ojalada	18.430	18.530
			Churra	18.430	18.480	Meat Lacaune	18.430	18.480
Regression top0.5% bracket 10 Mb	RegBrack10-CR1	2	Milk Lacaune	104.410		Ojalada	104.410	104.460
			Churra	104.460	104.510	Meat Lacaune	104.410	104.460
						Finnsheep	104.460	104.510
	RegBrack10-CR2	6	Milk Lacaune	34.875	38.875	Meat Lacaune	34.3747	38.175
			Comisana	36.125	38.325	Australian Poll Merino	35.525	38.225
	RegBrack10-CR3	20	Churra	49.971	50.171			
			Milk Lacaune	50.071				
Regression top0.5% bracket 20 Mb	RegBrack20-CR1	6	Milk Lacaune	34.825	38.525	Meat Lacaune	34.375	38.175
			Comisana	35.525	38.825	Australian Poll Merino	34.975	38.175

We also indicate if the same signature of selection was also identified in the non-dairy breeds.

The myostatin analysis suggested that a bracket size of 10 Mb was optimal for identification of selected region. However, because this is a new methodology, the results obtained for the dairy breeds with all three bracket sizes (5-, 10- and 20-Mb) were compared to aid interpretation of results based on this approach. The number of candidate regions identified in at least two dairy breeds decreased with increasing bracket size. For the 5-Mb bracket size, a total of seven candidate regions were observed, whereas only three and one candidate regions were observed for the 10- and 20-Mb bracket sizes, respectively (Table 3). The region commonly identified through the use of all three bracket sizes was located on OAR6 (RegBrack5-CR6, RegBrack10-CR2 and RegBrack20-CR1). The signal for this region was seen in Milk Lacaune (34.875–38.875 Mb, 10-Mb bracket) and Comisana (36.125–38.325 Mb, 10-Mb bracket) breeds. In addition, the Meat Lacaune variety also showed extreme results for this region for all three bracket sizes (34.375–38.175, 10-Mb bracket). Another region on OAR2 (104 Mb) was identified by both of the smaller bracket sizes.

Some of the inconsistencies between bracket sizes were investigated further. In several cases, where regions were not found in the top 0.5% of –log(p) values for a particular bracket size, they did appear in the top 1% of –log(p) values. Regarding the region on OAR20 (∼50 Mb) that was identified in two dairy breeds using the 10-Mb bracket size (RegBrack10-CR3, Table 3) but not using the 5-Mb bracket size: for Churra, positions within this region appeared within the top 1st percent of –log(p) values for the smaller bracket size but did not reach the threshold for the top 0.5th percent, whereas for Milk Lacaune, this region was identified using both bracket sizes. Regarding the five regions (Table 3) that were identified in two dairy breeds using 5-Mb bracket size but not 10-Mb, four of the regions were in the top 1st percent of –log(p) values for one or both of the dairy breeds. Two of these regions (RegBrack5-CR1 and RegBrack5-CR3) were found in Chios and Churra, however, while these regions were found for Churra using both the 5- and 10-Mb bracket sizes, for the 10-Mb bracket size, the top –log(p) values for Chios were dominated by regions on OAR13 and OAR16, which did not feature in the top –log(p) values for the other dairy breeds. Thus, these Chios-specific signals may have overwhelmed the more general dairy signals for the larger bracket size in this breed. The region labelled as RegBrack5-CR4, identified at ∼75 Mb on OAR3 for Churra and Milk Lacaune using the 5-Mb bracket size, did not feature in the top 1st percent of the –log(p) values for the 10-Mb bracket for either of these breeds. It is worth noting that regions identified using one bracket size but not a smaller one could reflect more recent selection events for which the pattern of heterozygosity with respect to distance from the selected locus appears linear rather than asymptotic in the smaller bracket.

### Convergence Candidate Regions (CCR)

Six candidate regions were detected in at least two breed pairs by the pair-wise F_ST_ comparison and in at least two breeds by a heterozygosity-based analysis (Table 4). One of the regions, CCR3 (OAR6∶30.367–41.863 Mb), was identified by all three analysis methods. The orthologous bovine genomic regions corresponding to each of the CCR are shown in Table 5. A total of 406 genes (positional candidate genes) were found in these six core regions (Table S3). There were three other regions where an F_ST_-CR signals was less than 1 Mb from an ObsHtz-CR signal (OAR3∶18.648–19.360 Mb, OAR3∶167.711–168.959 Mb, and OAR13∶95.801–98.865 Mb) but because they did not overlap, they were not considered as CCR.

**Table 4:** Convergence candidate regions (CCR) for selection signals identified for dairy sheep.

CCR	Chr.	Method	Individual methodcandidate region	Start marker*	Start position (Mb)	End marker*	End position (Mb)
CCR1	3	F_ST_	F_ST_-CR7	s51772	152.68	OAR3_165450843	154.582
		ObsHtz	ObsHtz-CR17	s26177	153.95	OAR3_165549468_X	154.679
CCR2	3	F_ST_	F_ST_-CR9	s34668	209.872	OAR3_234328134_X	215.814
		ObsHtz	ObsHtz-CR21	OAR3_229873996	211.624	s35739	215.403
CCR3	6	F_ST_	F_ST_-CR14	OAR6_34086500	30.367	OAR6_44210019	39.577
		Regression	RegBrack10-CR2	OAR6_38919831	34.875	OAR6_38919831	38.875
		ObsHtz	ObsHtz-CR27	OAR6_38585187	34.576	s38254	41.863
CCR4	13	ObsHtz	ObsHtz-CR42	OAR13_60893851	56.061	s63708	63.781
		F_ST_	F_ST_-CR24	s48133	62.277	OAR13_71091738	65.811
CCR5	15	F_ST_	Fst-CR26	s31340	72.774	OAR15_80448054	74.55
		ObsHtz	ObsHtz-CR44	s02793	72.843	s28875	72.948
CCR6	22	ObsHtz	ObsHtz-CR51	OAR22_23392099	19.588	OAR22_24747565	20.991
		F_ST_	F_ST_-CR28	OAR22_24682845	20.925	OAR22_26951573	23.157

A CCR region was defined when overlapping selection regions identified by the genetic differentiation analysis (in at least two breed pairs), averaged for a 9-SNP window size (F_ST_), and by at least one of the two heterozygosity-based analysis methodologies (in at least two breeds): observed heterozygosity, averaged for a 9-SNP window size (ObsHtz), and regression analysis, considering a 10-Mb bracket size (Regression).

^*^For Regression results, this indicates the closest marker to the Start/End position.

**Table 5:** Convergence candidate regions (CCR) for ovine dairy selection sweeps identified in this study.

Convergence candidate regions	Sheep genome range (Mb) (Oar v2.0)	Bovine genome range (Mb) (UMD 3.1)	Functional candidate genes based on Ogorevc et al. [22]	Other candidate genes^1^	QTL described in sheep	QTL described in cattle in relation to milk production and functional dairy traits (CattleQTLdb identifier^2^)	Nb. of positional candidates^3^
CCR1	OAR3∶152.680–154.679	BTA5∶46.720–49.009		*HMGA2*	Milk protein percentage [38]	Somatic cell score (2659), Milk fat yield (4495), Milk yield (2429), Rump length (3422), Stature (16277, 16278), Clinical mastitis (4973)	11
CCR2	OAR3∶209.872–215.814	BTA5∶106.976–112.636	*BID, MAFF, FKBP4, MKL1*	*CSNK1E*		Milk fat yield (daughter deviation) (9995), Milk protein yield (daughter deviation) (9994), Milk fat percentage (2717), Chest width (4623), Hip height (3420)	100
CCR3	OAR6∶30.367–41.863	BTA6∶31.710–43.022	*ABCG2, SPP1*	*FAM13A*		Milk protein percentage (EBV) (15002, 15003), Milk fat percentage (1753, 16057), Milk protein percentage (1755, 9913, 16058, 16059), Milk protein yield (daughter deviation) (10145), Milk fat yield (EBV) (11303, 12031), Milk protein yield (EBV) (11304), Milk yield (EBV) (11302), Somatic cell score (EBV) (6165, 6164), Milk fatty acid unsaturated index (11506, 11508, 11509, 11510), Milk myristoleic acid percentage (11507), Milk palmitoleic acid percentage (11505), Teat placement (10285), Udder attachment (10284)	32
CCR4	OAR13∶56.061–65.811	BTA13∶57.572–67.005	*POFUT1, TFAP2C, FAM110A, AHCY*	*GHRH, ASIP*		Milk protein percentage (2672), Milk protein yield (EBV) (6090), Milk fat yield (2555), Milk protein yield (daughter deviation) (10156), Milk protein yield (2671), Milk yield (2670), Udder attachment (1584), Udder composite index (1589), Udder depth (1588), Udder height (1586), Udder width (1587), Rump angle (3429), Foot angle (1583)	172
CCR5	OAR15∶72.774–74.550	BTA15∶75.154–76.879	*CD82*			Teat placement (1595), Udder cleft (1600), Udder composite index (1602), Milk fat yield (4503), Milk protein yield (4502), Milk proteinyield (EBV), (6103), Milk fat percentage (3452), Milk protein percentage (EBV) (11345), Milk yield (EBV) (11346)	17
CCR6	OAR22∶19.587–23.157	BTA26∶20.286–24.226	*CHUK, SCD*		Milk fatty acid composition [57], [58], Somatic cell score [60]	Milk yield (10452), Milk protein yield (10454), Milk fat yield (3636), Milk protein yield (3638), Milk protein yield (11702), Milk yield (11701), Milk fat yield (10453), Milk fat percentage (2598), Milk fat yield (2572), Milk protein yield (2573), Milk yield (2574), Milk protein percentage (4814), Milk protein percentage (3639), Milk yield (3634), Somatic Cell Count (1503)	74

The interval of each region (in bp) is based on the sheep genome reference sequence v2.0 (http://www.livestockgenomics.csiro.au/cgi-bin/gbrowse/oarv2.0/). The corresponding orthologous bovine genomic intervals are based on the bovine genome reference sequence UMD 3.1 (http://www.ensembl.org/Bos_taurus/Info/Index). The positional candidate genes that map within the bovine candidate range and that are included as candidate genes for milk production and mastitis traits in the database provided by Ogorevc et al. [22] are indicated as functional candidate genes. The affected trait and CattleQTLdb reference for previously reported cattle QTL that map within the bovine candidate genomic regions and that influence milk production traits or other functional traits related to dairy production are also indicated.

1 Other candidate genes. This category includes two types of genes:

–Those that although are not included in the Ogorevc et al. [22] database may be considered as candidate genes in relation to milk production related traits based on other studies.

–Those that are likely to be related to non-dairy selection signatures such as morphological traits and coat colour features.

2 CattleQTLdb identifier: Search reference number at http://www.animalgenome.org/cgi-bin/QTLdb/BT/search to find complete details about QTL reported in the orthologous region of the corresponding sheep CCR identified in this study.

3 Number of positional candidate genes extracted from the orthologous bovine region using BioMart for each of the labeled CCRs (based on Table S3).

Among the positional candidate genes extracted from the six CCRs, a search for functional candidates for milk production traits and mastitis was performed by comparison with the genes included in the Ogorevc et al. [22] database of cattle candidate genes for dairy-related traits. A total of 13 genes were common to these two lists (Table 5). The evidence for relationships with milk production traits for these genes was based on the different aspects considered in the Ogorevc et al. [22] database such as gene expression studies related to mammary gland (*TFAP2C, FAM110A, CD82, ABCG2*) or mastitis (*BID, MAFF, AHCY*), mouse model studies in which gene knockouts or expression of transgenes resulted in phenotypes associated with the mammary gland (*FKBP4, MKL1, POFUT1, CHUK*) and association studies of milk production traits (*ABCG2, SPP1, SCD*).

In order to assess whether there was greater overlap between the CCRs and candidate genes than expected by chance, we repeatedly (1 000 000 times) assigned regions of the same length as the CCR at random positions on the bovine genome and checked overlap with all candidate genes from the Ogorevc et al. [22] database that could be positioned on the bovine genome (423 genes). Although we could not do the test with the sheep genome as the annotation is not as complete, the length of the sheep and bovine genomes is very similar and so we expect this test would provide similar results. The number of overlaps between CCR regions and candidate genes based on a model with random positioning of CCR regions was very different from the actual situation: only 8.4% of the replicates contained any overlaps and the maximum number of overlaps was 4.

Some other positional candidate genes not included in the Ogorevc et al. [22] database were identified as possible functional candidates based on their known biological function and an exhaustive literature review of reported signatures of selection in dairy cattle (Table 5). There was also correspondence between the CCR and QTL previously reported in dairy cattle and sheep for milk production traits or functional traits related to dairy production (Table 5), which is discussed below.

## Discussion

This study reports the first genome-wide analysis of regions under selection for dairy traits in sheep. For this we have used the valuable information generated in the International Sheep HapMap project [14], through the use of the *Illumina OvineSNP50 BeadChip,* to evaluate a range of European sheep breeds that have been selected for dairy production. With the aim of identifying the signatures of selection specifically due to dairy selection and not related to other traits that may have been selection target in the studied sheep populations (e.g. coat colour), we also included in our study other non-dairy European sheep breeds. Furthermore, because of the difficulties in distinguishing between the effects caused in the genome by genuine selective sweeps rather than demographic events such as population expansion or contraction [16], we used three different analysis methods and only considered for further exploration those six regions identified by the F_ST_-based method and at least one of the two heterozygosity-based methodologies.

### Candidate Dairy Selection Regions

Based on the convergence among the three different analysis methods, six core regions were identified as candidate regions under positive selection in dairy sheep. Based on the comparison to predicted overlaps for randomly-positioned CCR, these regions were highly enriched for candidate dairy-related loci. We discuss further the CCR regions that meet specific criteria.

### Region Identified by all the Three Methods

**CCR3 (OAR6∶30.367–41.863 Mb).** The three analysis methods identified this region of positive selection in the first half of OAR6, which includes the *ABCG2* (ATP-binding cassette, sub-family G (white), member 2) and *SPP1* (osteopontin) genes (at 36.565–36.610 Mb and 36.708–36.720 Mb respectively), and is orthologous to the region of the bovine genome on BTA6 where several QTL for milk production traits have been reported (See Table 5 for QTL identifier number in the CattleQTLdb). This region also includes the *FAM13A* (family with sequence similarity 13, member A) gene, which has been shown to be associated with mastitis in Jersey cows [27]. In dairy cattle, strong selection signals have previously been identified [23], [24] in the proximity of the *ABCG2* gene, which harbors one of the few causal mutations or Quantitative Trait Nucleotide (QTN) described in livestock species [28]. In sheep, a selection signal in the *ABCG2* region has also been identified in a work focused on Altamurana sheep, where differences in allele frequencies were compared for animals with high and low milk yields [29].

The identification of a selection signature in this region of OAR6 by the pair-wise F_ST_ comparison (F_ST_-CR14) was based on four breed pairs. For the Milk Lacaune-Australian Poll Merino and the Comisana-Australian Poll Merino pairs, the signal of genetic differentiation involved the *ABCG2* and *SPP1* genes, whereas for the two other pairs, the identified signal was upstream (Chios-Sakiz; OAR6∶30.367–30.380 Mb) or downstream (Churra-Ojalada; OAR6∶39.316–39.577 Mb) of these genes. The ObsHtz analysis showed a selection signal (ObsHtz-CR27) for Milk Lacaune, Comisana and Churra dairy breeds, and also for three non-dairy breeds, Australian Poll Merino, Meat Lacaune and Ojalada. Both Lacaune breeds showed low values of ObsHtz extended for long intervals (3.48 and 5.47 Mb for Milk Lacaune and Meat Lacaune, respectively). With regard to the regression-based analysis, this region was the only one detected in multiple breeds for all three bracket sizes (for Milk Lacaune, Comisana, Meat Lacaune and Australian Poll Merino breeds).

Together these results suggest that CCR3 shows selection for dairy traits in several sheep breeds, and that this signal may be related to the documented effects of the *ABCG2* [28] or *SPP1* [30] genes on milk production and lactation regulation, respectively. The selection signal positioned directly at *ABCG2* and *SPP1* was only seen in the highly specialized breeds Milk Lacaune and Comisana (F_ST_, ObsHtz and Regression). In other dairy breeds for which the selection is more recent and less efficient (e.g. Churra and Chios), selection may not have substantially altered the frequencies of favoured alleles at these loci, which could explain why a strong selection signal directly at these genes was not observed. A previous study in Churra sheep found suggestive associations between the *ABCG2* gene and milk fat percentage and milk yield [31] while no studies to date have tested the effects of these two genes on dairy traits in the Lacaune and Comisana breeds.

The results reported in the current study also suggest that in this region of OAR6 there could be a selection signal related to meat specialized breeds such as Meat Lacaune, Australian Poll Merino and Ojalada. In this regard, it is worth noting that several QTL for growth and carcass traits have been described in the orthologous bovine region [32], [33]. Hence, analogous to the observations in the orthologous bovine region, this region of the sheep genome may influence both dairy and meat production traits.

### Regions with High F_ST_ in more than Two Breed Pairs

This criterion was used to highlight the CCR regions where the genetic differentiation analysis showed a particularly strong indication of a dairy selection signature, as this is possibly the most effective analysis performed in this study to detect regions specifically affected by dairy selection rather than selection acting on non-dairy-related traits. With the aim of establishing stringent criteria we consider in this section only those regions where more than two breed pairs (none sharing a common breed, as explained above) showed the selection signal. In addition to CCR3 discussed above, this category also includes the following two regions:

**CCR1 (OAR3∶152.680 to 154.679 Mb).** This core region, for which the F_ST_-selection signals were identified for the Churra-Ojalada, Comisana-Australian Poll Merino and East Friesian Brown-Finnsheep pairs, includes *HMGA2* (high mobility group AT-hook 2), a gene associated with human stature [34]. The identification of this gene as a selection target was also found in an analysis of dogs with divergent stature [10]. The bovine region orthologous to CCR1 includes QTL related to stature (with the *HMGA2* gene suggested as a possible causative locus [35]) and rump length (see Table 5). Hence, the CCR1 signal identified in the present study might indicate selection targeting sheep body conformation traits. This hypothesis would agree with the differences in body size between some of the pairs involved in this selection signal. For example, the adult weight of Australian Poll Merino is significantly higher than that of Lacaune and Comisana; Churra and East Friesian Brown are also generally heavier than their comparison breeds. *HGMA2* has also been suggested as a candidate gene related to ear size and shape in both pigs and dogs [36], [37], thus further investigation is required to assess whether there are differences in ear morphology between the sheep breeds showing this selection signal. Although the confidence interval of a QTL for protein percentage reported in Churra sheep [38] (Table 5) overlaps with CCR1, the causal mutation for that QTL was later found in the *LALBA* gene [39], which maps outside of this core region.
**CCR2 (OAR3∶209.872–215.814 Mb).** Four candidate genes in the orthologous bovine region to this CCR (distal end of BTA5) were identified from the Ogorevc et al. [22] database. Two of them were related to mastitis in a disease-induced mouse-model study [40]: *BID* (BH3 interacting domain death agonist), which is a pro-apoptotic induced gene, and *MAFF* (v-maf avian musculoaponeurotic fibrosarcoma oncogene homolog F), which is related to cell proliferation. The identification of two other genes as candidates for dairy traits in this regions, *FKBP4* (FK506 binding protein 4) and *MLK1* (mixed lineage protein kinase), was also based on mouse model studies (http://www.informatics.jax.org/). Furthermore, *FKBP4* is expressed in breast cancer tissue (Genes-to-Systems Breast Cancer database, G2SBC, http://www.itb.cnr.it/breastcancer//index.html) and *MLK1* is expressed in epithelial tumor cell lines of colonic, breast and esophageal origin [41]. QTL effects described in the bovine region orthologous to CCR2 (on BTA5) influence milk production and some conformation traits (Table 5). A previous study in dairy cows found a selection signature in this region [23]. In that case, the gene displaying the strongest evidence of selection was *CD163*, which is involved in the innate immune response and clearance of plasma hemoglobin [42]. This region also includes the gene coding for CSNK1ε (casein-kinase epsilon), which is related to circadian rhythms. In a study of the human milk fat globule transcriptome, *CSNK1ε* was identified as one of the nine core “clock” genes that showed differential expression over a 24-hour period time in lactating women [43]. Of particular interest is the finding that this OAR3 region was labelled as a CCR based on the overlap of candidate regions detected by pair-wise-F_ST_ in the pairs including the most highly specialized dairy breeds (Milk Lacaune, Comisana and East Friesian Brown), which may have been under selection for circadian-related adaptation of milk production to intensive milking.

### Other Regions

**CCR4 (OAR13∶56.061 to 65.811 Mb).** Several genes included in this core candidate region were also found in the Ogorevc et al. [22] database. The *POFUT1* (protein O-fucosyltransferase 1) gene plays a crucial role in Notch signaling, which regulates mammary stem cell function and luminal cell-fate commitment [44]. *TFAP2C* (transcription factor AP-2 gamma; activating enhancer binding protein 2 gamma) is involved in mammary development, differentiation, and oncogenesis playing a critical role in gene regulation in hormone responsive breast cancer [45], and *AHCY* (adenosylhomocysteinase) has been suggested as potentially involved in mastitis defense based on its disease-associated expression [46]. Another positional candidate gene for this core region is the *GHRH* (growth hormone-releasing hormone) gene. Although the direct relationship of this gene and milk production traits is still not clear [47], [48], its link to the somatotropic axis and other functional candidate genes included in the Ogorevc et al. [22] database (*GH*, *GHR*, *GHRHR*) suggest a possible influence, directly or indirectly, on dairy traits. In addition to these candidate dairy-related genes, the *ASIP* (Agoutí signaling protein) gene is also located in this region (OAR13∶63.028–63.033 Mb). This gene has a major role in metabolic processes [49] and coat colour pigmentation in mammalian species [50]. Based on the known associations between polymorphisms at this gene and coat colour patterns in sheep [51] it is possible that the identified selection signal results from coat colour selection. In their analysis of the complete HapMap dataset, Kijas et al. [14] also identified a selection signal near *ASIP.*
**CCR5 (OAR15∶72.774–74.550 Mb).** This region included the *CD82* (CD82 molecule) gene, which is included in the Ogorevc et al. [22] database based on its expression in the mammary gland. This gene is included in the group of genes that regulate breast cancer metastasis, as a metastasis suppressor [52]. Whereas no studies have reported an association of this gene with dairy related traits, there is a functional relationship between *CD82* and *ERBB3* (Receptor tyrosine-protein kinase erbB-3) [53], which is related to normal mammary development [54].
**CCR6: (OAR22∶19.588–23.157 Mb).** Two functional candidate genes [22] were found in this region: *SCD* (Stearoyl-CoA desaturase) and *CHUK* (conserved helix-loop-helix ubiquitous kinase). The *SCD* gene encodes a multifunctional complex enzyme important in the cellular biosynthesis of fatty acids. Several studies in different populations of dairy cattle have reported associations between polymorphisms at this gene with milk production traits [55] and milk fatty acid composition [56]. In sheep, the *SCD* gene has been suggested as positional and functional candidate gene for a QTL identified on OAR22 in a Sarda × Lacaune back-cross population for the ratio of conjugated linoleic acid to vaccenic acid in sheep milk [57]. A later study in Churra sheep also identified a QTL on OAR22 for the same trait close to the *SCD* position, although various analyses questioned this gene as responsible for the identified effect [58]. The *CHUK* gene is listed in the Ogorevc et al. [22] database because it is expressed in breast cancer tumors and is a regulator of mammary epithelial proliferation [59]. According to the SheepQTL database, this region includes a QTL for somatic cell score described in an Awassi x Merino cross population [60] and it has also been identified as a selection signal by the analysis of allele frequency differences between animals with divergent milk yields reported in Altamurana sheep [29].

The bovine region orthologous to CCR6 (on BTA26), overlaps with a region showing a selection signature in dairy cattle [23], where the *C10ORF76* (chromosome 10 open reading frame 76) gene was associated with the strongest selection signal. Although there is not a reported association of this gene with milk production traits, it is expressed in the mammary gland and it is altered in breast cancer cells, based on the G2SBC database.

### Inconsistencies between this Study and Previous QTL and Selection Mapping Studies of Cattle and Sheep

Although all six CCR overlapped with QTL for dairy traits in sheep or cattle (Table 4 and discussed above), our study did not identify a selection signal close to several genes previously associated with dairy traits in sheep and cattle. For example, there were no CCR near the *LALBA* (alpha-lactalbumin) gene (OAR3∶137 Mb), where a particular variant has been recently been proposed to explain a QTL for milk protein percentage identified in Churra sheep [39]. The lack of signal near this QTL in the F_ST_ analysis of Churra vs Ojalada is consistent with the fact that the causative mutation is still segregating in Churra, which allowed its identification as QTL.

In addition, in their analysis of the complete Sheep HapMap dataset, Kijas et al. [14] reported positive selection surrounding the *PRLR* gene, which is associated with milk traits in dairy cattle [61]. In our study, although none of the CCRs map to OAR16, where this gene is located (39.250–39.284 Mb), it is worth mentioning that this gene is included in the interval of F_ST_-CR27 (OAR16∶37.347–40.850 Mb), which was identified based on the signals detected in three breed pairs involving the most specialized dairy breeds in this study (East Friesian Brown-Finnsheep, Milk Lacaune-Australian Poll Merino and Comisana-Australian Poll Merino) but was not classified a CCR due to the lack of selection signals from the heterozygosity-based methods. Other regions that were detected by the F_ST_-pairwise comparison for many breed pairs but that were not supported by the heterozygosity-based methods were found on OAR2 (F_ST_-CR2∶52.346–53.409 Mb) and OAR9 (57.363–60.849 Mb). Whereas the first region does not include any functional candidate gene for dairy traits, the region in OAR9 included three genes related to the metabolism of fatty acids (*FABP4, FABP5* and *FABP9*). *FABP4* and *FABP5* have been shown to be highly expressed in the mammary gland during lactation [62] and significant associations have been found between *FABP4* and fatty acid composition of bovine milk [63]. We acknowledge that one or more of these regions may represent false negatives that were missed by our stringent selection signal criteria. However, because of the difficulty in linking a sweep signal to a given phenotype, we suggest that application of stringent criteria in this type of study is an appropriate option to avoid reporting long lists of candidate regions based on spurious results.

We also did not find evidence of selection on some major candidate genes for milk production for which selection signatures have been observed in cattle (e.g. *DGAT1:* OAR9∶13.534–13,543 Mb; *GHR:* OAR16∶32.068–32.231). In contrast to our results, the *GHR* gene (BTA20) showed the largest difference in sliding window average allele frequencies in a study of divergent selection between dairy and beef cattle [24], and also showed significant extended haplotype homozygosity [25]. With regard to *DGAT1*, evidence of selection has also been identified when comparing dairy and beef cattle breeds [24].

In their study, Kijas et al. [14] also identified a strong selection signal on OAR10, associated to the presence or absence of horns and close to the gene responsible of the polled phenotype, *RXFP2* (relaxin/insulin-like family peptide receptor 2) gene (OAR10∶27.602–27.646 Mb). In our study, a selection signal was identified in this region based on the ObsHtz-based method (ObsHtz-CR33∶24.856–27.897 Mb, for the dairy breeds Comisana, Churra and Milk Lacaune) and the F_ST_-based method (OAR10∶25.540–28.983 Mb). However because the F_ST_ signal was due only to the two breed pairs involving the Australian Poll Merino, this was not labelled as F_ST_-CR.

Apart from the overlap between two CCRs (CCR3 on OAR6 and CCR6 on OAR22) with the selection signals identified in Altamurana sheep for milk yield [29], we did not find evidence of selection near the signals reported for this Italian breed. This lack of correspondence may derive from breed-specific signals reported for Altamurana.

### Comparison of the Three Selection Mapping Methods

From our point of view, the analysis method that involved the estimation of pair-wise F_ST_ for pairs of related breeds showing divergent specialization (one for milk production, one not) should be the most powerful analysis in terms of identifying selection specifically related to dairy production. Four out of the 28 candidate regions showing multiple pair-wise F_ST_ signals were detected in four out of the six breed pairs (F_ST_-CR3, F_ST_-CR9, F_ST_-CR14 and F_ST_-CR18). Of these, F_ST_-CR3 (OAR2∶52.346–53.409 Mb) was not included as a CCR due to the lack of consistency with the ObsHtz or 10-Mb Regression analysis results, although the same region was identified by the 5-Mb Regression analysis (RegBrack5-CR1 in Table 3) in two dairy breeds (Churra and Chios) and one non-dairy breed (Ojalada). Given that no functional candidate genes from the Ogorevc et al. [22] database were found in the orthologous bovine region, it is possible that this region underlies breed differentiation not directly related to dairy traits.

Among the 55 candidate regions identified based on the ObsHtz analysis (ObsHtz-CRs, Table S2), there were only twelve regions showing a signal in dairy but not in non-dairy breeds (ObsHtz-CR3, 7, 8, 10, 12, 25, 28, 29, 31, 41, 45 and 55). Considering that the background genome has been previously selected for meat, maternal characteristics, and other traits, whereas the development of dairy breeds is much more recent, it would be expected that the selection signals specifically related to dairy traits would not be seen in the other breeds (although Meat Lacaune could be an exception). However, as none of these regions showing a reduction of heterozygosity exclusively in dairy breeds were identified by the F_ST_-based method, they were not identified as final core CCRs (and thus are not present in Table 4). Although the evidence linking these regions to dairy-related selection is weaker than for the CCRs, we performed an additional search for functional candidate genes and dairy-related QTL mapping within these regions, similar to that performed in the eight identified CCRs (see Table S4). A total of 118 genes were extracted from the orthologous bovine regions of these eleven dairy-breed-limited regions of reduced heterozygosity (data not shown). Among them, only the *HSPD1* (Heat shock 60 kDa protein 1; chaperonin) gene is included in the Ogorevc et al. [22] database, due to its expression in the mammary gland. This gene is also included in the G2SBC database although no studies have reported so far its association with milk production traits. Interestingly, among the dairy QTL detected in these regions there is greater overlap with ovine QTL for milk production traits (Table S4) than for the list of core CCRs. Hence, these regions identified exclusively by ObsHtz could include gene variants occurring in individual dairy breeds, as it is the case for many of the QTL described in sheep.

There were eight regions that overlapped between those identified by F_ST_ (including a full set of regions, including those that contained pairs with the same breeds that were removed from Table S1) and ObsHtz (out of 35 and 55, respectively). The explanation for the higher number of regions identified by ObsHz is that the regions identified using F_ST_ were slightly larger (incorporated more windows) than those identified using ObsHtz.

There were far fewer signals identified using the Regression approach than either F_ST_ or ObsHtz. Although the top (or bottom) 0.5th percent results were considered as signals of selection for all methods, the Regression method first filtered out the intervals with non-significant and non-asymptotic regression patterns, and thus the total number of eligible intervals was substantially reduced compared to the other approaches in which the distribution of F_ST_/ObsHtz values for all markers (with the exception of those on the very ends of the chromosomes) was considered. Thus the implementation of Regression in this study was more stringent than the other methods.

The regions identified by the Regression method showed greater overlap with ObsHtz than F_ST_, which is not surprising since both Regression and ObsHtz are designed to detect regions with a reduction in diversity. For the 10-Mb bracket size (results considered for the identification of CCR), all three regions identified with the Regression approach overlapped with those identified with ObsHtz while one out of the three, RegBrack10-CR2, overlapped with the regions identified with F_ST,_ and was therefore considered as CCR (CCR3).

## Conclusions

The results reported here provide a genome-wide map of selection signatures in the dairy sheep genome. The six core candidate regions identified are likely to influence traits of economic interest in dairy sheep production and can be considered as starting points for future studies aimed at the identification of the causal genetic variation underlying these signals. For some of these regions, strong candidate genes have been proposed (e.g. *ABCG2, SPP1*), whereas some other genes designated as candidates based on their association with sheep and cattle dairy traits (e.g. *LALBA, DGAT1A*) were not associated with a detectable sweep signal. Discrepancies between selection signals in dairy sheep and cattle may be explained either by statistical or biological factors, such as the limited statistical power of the analyses to identify effects of small magnitude or the fact that the genetic architecture of milk production and dairy-related traits substantially differs from sheep to cattle and also between the different breeds of dairy sheep, which have been subjected to different levels of selection pressure. Many of the identified regions corresponded to orthologous regions in cattle where QTL for dairy traits have been identified. Due to the limited number of QTL studies reported in sheep compared with cattle, the results illustrate the potential value of the study of selection signatures to uncover mutations with potential effects on quantitative dairy sheep traits. Additional studies are needed to confirm and refine the results reported here. To this end, the recent availability of the high-density ovine chip (700 K) will provide a valuable tool to perform more powerful and precise selection mapping studies.

## Supporting Information

Table S1 Candidate regions for signatures of selection identified on the basis of the pair-wise F_ST_ analysis.
(PDF)

Table S2 Candidate regions identified based on reduced heterozygosity signals identified in at least two of the dairy breeds.
(PDF)

Table S3 List of all genes from the orthologous bovine genome regions corresponding to the six convergence candidate regions (CCR) for dairy selection sweeps identified in this study, extracted using the Biomart tool (http://www.biomart.org/).
(XLSX)

Table S4 Candidate regions identified by the analysis based on observed heterozygosity (ObsHtz-CR), averaged in sliding windows of 9 SNPs (ObsHtz-9SNPW), that were exclusively detected in dairy breeds.
(PDF)

File S1 **Summary of the criteria for selection of breeds to be included in the study, including the results of a Principal Component Analysis (PCA) performed with the initial set of breeds considered.**
(PDF)

File S2 **Summary of the results of the analysis performed in this work in relation to the myostatin (** ***GDF-8*** **) gene region.** These results were evaluated to establish criteria for the analyses performed to detect dairy selection signatures in the dairy breeds.
(PDF)
