# Supplementary material for: Application of Selection Mapping to Identify Genomic Regions Associated with Dairy Production in Sheep
Source: PLoS One. 2014 May 1;9(5):e94623. doi: 10.1371/journal.pone.0094623 (PMC4006912; doi:10.1371/journal.pone.0094623)
Supplement: Table S1 — Candidate regions for signatures of selection identified on the basis of the pair-wise FST analysis. (PDF) [file pone.0094623.s001.pdf]

Table S1 for

Application of selection mapping to identify genomic regions associated with dairy production in sheep

Authors: Beatriz Gutiérrez-Gil[1]*, Juan Jose Arranz[1], Ricardo Pong-Wong[2], Elsa García-Gámez[1], James Kijas[3], Pamela Wiener[2]

Table S1. Candidate regions for signatures of selection identified on the basis of the pairwise F_{ST} analysis performed on six dairy–non-dairy breed pairs. For each dairy-non-dairy breed pair, the results contain the top 0.5th percent of the distributions of F_{ST} values, averaged in sliding windows of 9 SNPs. Only the regions that were found in at least two pairs of breeds (that did not have a breed in common) were labeled as F_{ST}-based candidate regions (F_{ST}-CRs). Gaps up to 2 Mb were allowed between positions identified within the same breed pair and between different breed pairs to consider a single selection signal. Positions of the markers (Mb) are referred to the Sheep Genome Assembly v2.0 (update September 2011).

Fst-Candidate region (Fst-CR)	Chr	Breed Pair	Start marker	Start position (Mb)	End marker	End position (Mb)
Fst-CR1	1	Churra-Ojalada	OAR1_110170625	102.467714	s70081	102.594066
		Comisana-Australian Poll Merino	OAR1_112076929	104.214069	OAR1_112239116	104.372893
		Chios-Sakiz	s11941	105.338083	OAR1_113402761	105.563436
Fst-CR2	1	Milk Lacaune-Meat Lacaune	OAR1_245302998	228.057561	OAR1_247070566	229.569756
		East Friesian Brown-Finnsheep	OAR1_245583569	228.280721	OAR1_249572898	231.993714
Fst-CR3	2	Churra-Ojalada	s60207	52.34599	OAR2_55861669	52.563013
		Milk Lacaune-Meat Lacaune	OAR2_55752685	52.4461		
		Comisana-Australian Poll Merino	OAR2_56432170	52.885699		
		Chios-Sakiz	OAR2_56768579	53.239006	s23499	53.409079
Fst-CR4	2	Comisana-Australian Poll Merino	OAR2_87854424	83.150938	OAR2_90110357	85.329228
		Chios-Sakiz	OAR2_87942973	83.248507		
Fst-CR5	3	East Friesian Brown-Finnsheep	s65219	18.951292	s05622	19.360373
		Milk Lacaune-Australian Poll Merino	s13092	19.188714	s05622	19.360373
Fst-CR6	3	East Friesian Brown-Finnsheep	OAR3_84348827	79.410427		
		Chios-Sakiz	s63332	79.545868	OAR3_84689579	79.728228
Fst-CR7	3	Churra-Ojalada	s51772	152.679607	OAR3_163641518	153.007357
		Comisana-Australian Poll Merino	OAR3_164170826	153.444621	OAR3_165450843	154.582317
		East Friesian Brown-Finnsheep	OAR3_164788310	154.01323	OAR3_165060142	154.207199
Fst-CR8	3	East Friesian Brown-Finnsheep	OAR3_180416904	167.711074	OAR3_180484085	167.782385
		Milk Lacaune-Australian Poll Merino	OAR3_180416904	167.711074	OAR3_180647061	167.92624
Fst-CR9	3	Milk Lacaune-Meat Lacaune	s34668	209.872091	s08021	210.525298
		Milk Lacaune-Australian Poll Merino	s14713	211.970434	s36662	212.063962
		Comisana-Australian Poll Merino	s26777	213.468361	s38745	213.645308
		East Friesian Brown-Finnsheep	s68727	215.304893	OAR3_234328134_X	215.814066
Fst-CR10	4	Chios-Sakiz	OAR4_59113372	55.121399		
		Milk Lacaune-Meat Lacaune	s04516	55.485954	s32998	55.645159
Fst-CR11	4	Chios-Sakiz	s64477	72.568414	OAR4_79957476	74.701765
		East Friesian Brown-Finnsheep	s12823	74.234142		
Fst-CR12	4	Milk Lacaune-Meat Lacaune	OAR4_85593968	80.007532	OAR4_90286613_X	84.344311
		East Friesian Brown-Finnsheep	OAR4_88152650	82.245971	OAR4_88330712	82.403195
Fst-CR13	4	Milk Lacaune-Australian Poll Merino	OAR4_102186070	95.801069	OAR4_102504729	96.11515
		Milk Lacaune-Meat Lacaune	OAR4_102230122	95.85043	OAR4_102402825	96.019593
		Churra-Ojalada	s44110	97.181846		
Fst-CR14	6	Chios-Sakiz	OAR6_34086500	30.366856	OAR6_34099005	30.37978
		Milk Lacaune-Australian Poll Merino	OAR6_34609120	30.760292	OAR6_43064935	38.243473
		Comisana-Australian Poll Merino	OAR6_38546054	34.536979	OAR6_42185110	37.494544
		Churra-Ojalada	OAR6_43842580	39.316435	OAR6_44210019	39.577446
Fst-CR15	6	Comisana-Australian Poll Merino	OAR6_76284920	69.987431	s13781	70.599139
		Chios-Sakiz	OAR6_78103269	71.885158		
Fst-CR16	7	Chios-Sakiz	OAR7_57131990	51.584028	OAR7_57384468	51.805664
		East Friesian Brown-Finnsheep	OAR7_57594015	52.07214		
Fst-CR17	8	East Friesian Brown-Finnsheep	OAR8_34133754	31.505215	s50528	32.978156
		Churra-Ojalada	s50528	32.978156	OAR8_35827974	33.178871
Fst-CR18	9	Milk Lacaune-Meat Lacaune	OAR9_60243687	57.362506		
		Chios-Sakiz	s71892	59.029356		
		Milk Lacaune-Australian Poll Merino	s13022	60.567978	OAR9_63903278	60.821175
		Comisana-Australian Poll Merino	s57864	60.609845	OAR9_63720685	60.628768
		Churra-Ojalada	OAR9_63928272	60.846404		
Fst-CR19	9	Chios-Sakiz	OAR9_72753560	68.819131		
		East Friesian Brown-Finnsheep	OAR9_72753560	68.819131	OAR9_72793362	68.85534
		Churra-Ojalada	OAR9_73519625	69.587011	s46550	69.830122
Fst-CR20	10	Churra-Ojalada	OAR10_38873282	36.309562	OAR10_39526423_X	36.868674
		East Friesian Brown-Finnsheep	OAR10_39356591	36.711162	OAR10_40078675	37.480614
Fst-CR21	11	Milk Lacaune-Meat Lacaune	s43916	33.918454	s37019	36.506595
		Milk Lacaune-Australian Poll Merino	OAR11_36358079	34.118663	s33971	34.163747
		Chios-Sakiz	OAR11_37440114	35.253844		
Fst-CR22	12	Milk Lacaune-Australian Poll Merino	OAR12_8518601	6.113085	s02824	6.314395
		Churra-Ojalada	OAR12_10315606	7.84721	OAR12_10373193	7.894632
Fst-CR23	13	Milk Lacaune-Meat Lacaune	s27419	48.971612	OAR13_56607666	52.062577
		Comisana-Australian Poll Merino	OAR13_55596698	51.039293	OAR13_55835685	51.277367
		Milk Lacaune-Australian Poll Merino	s67173	52.777853	OAR13_57596022	52.946055
Fst-CR24	13	Comisana-Australian Poll Merino	s48133	62.276683	OAR13_71091738	65.811237
		Churra-Ojalada	OAR13_69221369_X	63.911086	s17594	64.053077
Fst-CR25	15	Churra-Ojalada	s64562	16.673108	OAR15_21513878	20.687439
		East Friesian Brown-Finnsheep	s28843	21.72601		
Fst-CR26	15	Comisana.vs.AustralianPollMerino	s31340	72.774077	s73092	72.88226
		Chios.vs.Sakiz	OAR15_80448054	74.499334	s72518	74.550363
Fst-CR27	16	East Friesian Brown-Finnsheep	OAR16_40313142	37.347459	OAR16_44102697	40.850203
		Milk Lacaune-Australian Poll Merino	OAR16_42131900	39.068289	s15191	39.263122
		Comisana-Australian Poll Merino	OAR16_44680056	40.165782	OAR16_44884811	40.330712
Fst-CR28	22	Chios-Sakiz	OAR22_24682845	20.925489	OAR22_24815596	21.058183
		East Friesian Brown-Finnsheep	OAR22_26951573	22.959209	s67752	23.157094
