# Supplementary material for: Application of Selection Mapping to Identify Genomic Regions Associated with Dairy Production in Sheep
Source: PLoS One. 2014 May 1;9(5):e94623. doi: 10.1371/journal.pone.0094623 (PMC4006912; doi:10.1371/journal.pone.0094623)
Supplement: Table S2 — Candidate regions identified based on reduced heterozygosity signals identified in at least two of the dairy breeds. (PDF) [file pone.0094623.s002.pdf]

Table S2 for

Application of selection mapping to identify genomic regions associated with dairy production in sheep

Authors: Beatriz Gutiérrez-Gil[1]*, Juan Jose Arranz[1], Ricardo Pong-Wong[2], Elsa García-Gámez[1], James Kijas[3], Pamela Wiener[2]

Table S2. Candidate regions identified based on reduced heterozygosity signals identified in at least two of the analyzed dairy breeds. Non-dairy breeds showing a coincident reduced heterozygosity signal are also indicated. For each breed, the results contain the bottom 0.5th percent of the distributions of observed heterozygosity values, averaged in sliding windows of 9 SNPs. Only the regions that were found in at least two dairy breeds were labeled as candidate regions based on the reduction of observed heterozygosity (ObsHtz-CRs). Gaps up to 2 Mb were allowed between positions identified within the same breed and between different breeds to consider a single selection signal. Positions of the markers (Mb) are referred to the Sheep Genome Assembly v2.0 (update September 2011).

ObsHtz-Candidate region (ObsHtz-CR)	Chr	Dairy breeds					Non-dairy breeds				
		Breeds	Start marker	Start position (Mb)	End marker	End position (Mb)	Breeds	Start marker	Start position (Mb)	End marker	End position (Mb)
Obs_Htz1	1	Milk Lacaune	OAR1_137761966	127.147272	OAR1_137785444	127.181978	Finnsheep	OAR1_138135147	127.487148	s13122	127.757038
		Churra	OAR1_138170073	127.525829	OAR1_138302052	127.655271					
Obs_Htz2	1	Comisana	s43775	191.715311	s28201	191.726576	Australian Poll Merino	s03519	191.732813		
		Chios	s73989	191.896602	OAR1_207015625_X	191.993294					
Obs_Htz3	2	Comisana	OAR2_20411070	20.132217	OAR2_20557855	20.27409					
		Churra	OAR2_20494849	20.200641							
Obs_Htz4	2	Comisana	OAR2_76331680	72.044265	OAR2_79499240	75.030746	Finnsheep	OAR2_75832730	71.577808	OAR2_75908169	71.612583
		East Frisian Brown	OAR2_77962229	73.59807	OAR2_78106813	73.747233	Meat Lacaune	OAR2_77568893	73.183462	OAR2_77690140	73.288963
							Australian Poll Merino	OAR2_77705450	73.305483	s59857	73.888851
Obs_Htz5	2	Milk Lacaune	s41033	104.332992	OAR2_111932274	104.586592	Meat Lacaune	s41033	104.332992	OAR2_111932274	104.586592
		Churra	s22902	104.395536	s67874	104.642351	Finnsheep	s22902	104.395536	s67874	104.642351
							Ojalada	s22902	104.395536	s29891	104.501488
							Australian Poll Merino	s29891	104.501488		
Obs_Htz6	2	Churra	OAR2_130265015	122.212699	OAR2_130817865	122.86725	Ojalada	OAR2_130413374	122.472584	OAR2_130656808	122.697031
		Milk Lacaune	OAR2_130413374	122.472584	OAR2_130656808	122.697031					
		Comisana	s18930	122.622313	s46322	123.095079					
		Chios	OAR2_130656808	122.697031	OAR2_131507986	123.721808					
Obs_Htz7	2	East Frisian Brown	OAR2_190455427	180.309886	OAR2_192112657	181.952078					
		Churra	OAR2_193567149	183.403726							
Obs_Htz8	2	Chios	OAR2_210373144	199.479344	OAR2_210391244	199.493856					
		Milk Lacaune	s70318	199.813655							
Obs_Htz9	2	Chios	OAR2_222355235	211.20491			Ojalada	OAR2_222327692	211.177754		
		Comisana	OAR2_222355235	211.20491			Australian Poll Merino	OAR2_222518452	211.36389		
Obs_Htz10	2	Milk Lacaune	OAR2_244597797	232.586994	OAR2_244925652	232.809436					
		Churra	s01478	234.42203	OAR2_246577997	234.454459					
Obs_Htz11	2	Churra	OAR2_252085914	239.683321	s04809	241.810875	Meat Lacaune	OAR2_252367108	239.948483	s00772	241.977125
		Comisana	s68726	241.681223	s72745	241.755749	Australian Poll Merino	s58181	240.928078	s04809	241.810875
		Milk Lacaune	s68726	241.681223	s64586	241.79047	Finnsheep	s10651	241.717335		
							Ojalada	s10651	241.717335	s43939	241.929821
Obs_Htz12	3	Churra	s46658	18.647955							
		Milk Lacaune	s46658	18.647955	s00385	18.858614					
Obs_Htz13	3	Milk Lacaune	OAR3_34159096	31.475728	s67270	32.053944	Meat Lacaune	OAR3_34159096	31.475728	OAR3_34470839	31.786813
		East Frisian Brown	OAR3_34209284	31.520076	s67270	32.053944	Ojalada	OAR3_34209284	31.520076		
		Comisana	OAR3_34470839	31.786813	s67270	32.053944	Finnsheep	OAR3_35192406	32.520276		
Obs_Htz14	3	Milk Lacaune	OAR3_79812234	75.12407	s52383	75.334539	Finnsheep	s40967	75.200035		
		Chios	s40967	75.200035	OAR3_79936157	75.239426					
		Churra	s40967	75.200035	OAR3_80038014_X	75.340524					
Obs_Htz15	3	Comisana	OAR3_127437500	119.375297	OAR3_127719431	119.73187	Ojalada	OAR3_128558246	120.420761		
		Chios	s70128	119.623504	s70128	119.623504					
		East Frisian Brown	OAR3_127924329_X	119.921829	OAR3_127956387_X	119.9636					
Obs_Htz16	3	East Frisian Brown	OAR3_134591031	125.994367	OAR3_134708445	126.116303	Ojalada	OAR3_132722549	124.350223	OAR3_132876525	124.473799
		Chios	OAR3_134841132	126.252978							
Obs_Htz17	3	Comisana	s26177	153.950444	OAR3_165549468_X	154.679398	Meat Lacaune	OAR3_165050963	154.197278	OAR3_165060142	154.207199
		Milk Lacaune	OAR3_165060142	154.207199	OAR3_165200988	154.347363	Sakiz	OAR3_165050963	154.197278	OAR3_166244306	155.291702
Obs_Htz18	3	Churra	s75549	163.340446	s28012	163.447575	Sakiz	s35593	162.838475	s03123	163.200412
		Milk Lacaune	OAR3_175355785	163.685017			Finnsheep	OAR3_174446873	163.148256		
							Meat Lacaune	OAR3_175355785	163.685017		
Obs_Htz19	3	Churra	OAR3_181499831	168.655327	OAR3_181815353	168.958984	Australian Poll Merino	OAR3_181535066	168.688319	OAR3_181815353	168.958984
		Milk Lacaune	OAR3_181499831	168.655327	OAR3_181815353	168.958984	Meat Lacaune	OAR3_181535066	168.688319	OAR3_181714610	168.866473
		East Frisian Brown	OAR3_181535066	168.688319	OAR3_182492456	169.636983	Finnsheep	OAR3_181576673	168.751495	OAR3_181714610	168.866473
		Comisana	OAR3_181576673	168.751495	OAR3_181714610	168.866473	Ojalada	OAR3_181576673	168.751495	s36931	170.561972
							Sakiz	OAR3_181688249	168.83807	OAR3_181815353	168.958984
Obs_Htz20	3	Churra	OAR3_196791000	182.497709			Meat Lacaune	OAR3_196913312	182.643043		
		Milk Lacaune	OAR3_196913312	182.643043	OAR3_197402139	183.099123					
Obs_Htz21	3	Chios	OAR3_229873996	211.624402	s22341	211.87793	Australian Poll Merino	s41320	211.806587		
		Churra	s17644	212.763057	s66905	213.048035	Ojalada	DU384041_287	212.622841	s23020	212.887492
		East Frisian Brown	OAR3_233491871	214.981744	s35739	215.4033					

Obs_Htz22	4	Churra	OAR4_11306223	10.518307	OAR4_12079184	11.293408	Meat Lacaune	OAR4_12079184	11.293408		
		Comisana	OAR4_12000546	11.218811			Ojalada	OAR4_12079184	11.293408		
Obs_Htz23	4	Comisana	s28608	97.763212	OAR4_105812413	98.865276	Australian Poll Merino	s16828	97.812402	s37018	97.871965
		Churra	s16828	97.812402	s37018	97.871965	Ojalada	s37018	97.871965		
Obs_Htz24	4	Milk Lacaune	s06803	112.216316	OAR4_120520710	112.268889	Finnsheep	s56814	113.730938		
		Churra	OAR4_121859278	113.688324							
Obs_Htz25	5	Chios	OAR5_43897574	40.407364	s72060	41.107358					
		Milk Lacaune	s74709	40.894605							
Obs_Htz26	6	Milk Lacaune	OAR6_26894928	23.623375			Meat Lacaune	OAR6_26396811	23.210527	OAR6_26894928	23.623375
		Chios	s17623	25.885521	OAR6_29577816	25.949127	Ojalada	OAR6_26396811	23.210527	OAR6_31567469	27.819055
							Australian Poll Merino	OAR6_31621001	27.872898		
							Sakiz	OAR6_33453920	29.69792		
Obs_Htz27	6	Milk Lacaune	OAR6_38585187	34.57594	OAR6_42834740	38.054381	Australian Poll Merino	OAR6_36395028	32.401102	OAR6_46589899	41.873949
		Comisana	OAR6_41044118	36.436857	OAR6_42317192	37.632679	Meat Lacaune	OAR6_38383335	34.365051	OAR6_44473865	39.833644
		Churra	s16069	41.629024	s38254	41.862923	Ojalada	s43499	36.056663	OAR6_48259046	43.363614
Obs_Htz28	7	Churra	OAR7_19866890_X	19.072325	s68550	19.257574					
		Milk Lacaune	OAR7_19928730	19.134616							
Obs_Htz29	7	Comisana	OAR7_46818598	42.026482							
		Chios	OAR7_47960594	43.116197	s68972	43.570232					
Obs_Htz30	7	Milk Lacaune	OAR7_60354601	54.41476	s30690	58.162605	Ojalada	OAR7_60354601	54.41476	OAR7_66685731	60.560526
		Chios	OAR7_65038832	59.047277	OAR7_65338086	59.341935	Meat Lacaune	OAR7_62610703	56.372667	OAR7_65264623	59.275972
		Churra	OAR7_65120201	59.12761			Australian Poll Merino	s00240	58.138119	s51528	60.408964
		Comisana	OAR7_66489587	60.45256	OAR7_66685731	60.560526	Finnsheep	s30690	58.162605	OAR7_64371135	58.364778
							Australian Poll Merino	OAR7_64280352	58.272169	s51528	60.408964
							Sakiz	OAR7_64854028	58.864481	OAR7_66229320	60.192716
Obs_Htz31	8	Milk Lacaune	OAR8_83750883	78.04147							
		East Frisian Brown	s34293	78.728134	s66278	78.759335					
Obs_Htz32	9	Milk Lacaune	s09014	74.560664	s39785	74.609642	Australian Poll Merino	s09014	74.560664		
		Chios	OAR9_80693962	76.140824	OAR9_80804089	76.242733	Meat Lacaune	s09014	74.560664		
							Ojalada	s22340	74.659349		
							Sakiz	OAR9_80534807_X	75.985327	OAR9_81054740_X	76.651847
Obs_Htz33	10	Comisana	OAR10_26652355	24.855758	s38815	28.800676	Finnsheep	OAR10_27085156	25.261864	OAR10_29546872	27.557292
		Churra	OAR10_28646479	26.67809	OAR10_29737372	27.837794	Meat Lacaune	OAR10_29448537	27.460172	OAR10_29737372	27.837794
		Milk Lacaune	OAR10_29389966_X	27.398222	OAR10_29793750	27.896977	Ojalada	OAR10_29538398	27.547387	OAR10_30790959	28.876748
Obs_Htz34	10	Comisana	OAR10_44470993	42.082634	OAR10_44655795	42.272772	Finnsheep	OAR10_43391432	40.671337	OAR10_45065959	42.682521
		Chios	OAR10_44655795	42.272772	OAR10_44834335	42.474049	Ojalada	OAR10_44470993	42.082634	OAR10_44834335	42.474049
		Milk Lacaune	OAR10_44655795	42.272772	OAR10_44834335	42.474049	Sakiz	OAR10_44470993	42.082634	OAR10_44615697	42.230982
							Meat Lacaune	OAR10_44509259	42.123799	OAR10_44834335	42.474049
Obs_Htz35	11	Churra	OAR11_18701428	18.346557	s69909	18.602308	Meat Lacaune	s13840	16.449218	s27474	18.671334
		Milk Lacaune	OAR11_18701428	18.346557	s27474	18.671334	Ojalada	OAR11_18701428	18.346557	s69909	18.602308
		Comisana	OAR11_18815864	18.454208	OAR11_18909056	18.544951	Australian Poll Merino	OAR11_18815864	18.454208	OAR11_19810690	19.403144
							Sakiz	OAR11_19810690	19.403144		
Obs_Htz36	11	East Frisian Brown	s59074	22.135921	OAR11_23034869	22.199593	Meat Lacaune	s51197	22.183054	s43495	22.368448
		Churra	s51197	22.183054							
		Milk Lacaune	s51197	22.183054	s43495	22.368448					
Obs_Htz37	12	Comisana	s13858	24.480926			Sakiz	OAR11_26873926	25.777153	s31301	26.612875
		Churra	s14394	24.545136	s43122	24.803153	Australian Poll Merino	s51636	26.318526	s23362	29.221714
		Chios	s10340	24.60133	s62452	29.530127					
		Milk Lacaune	OAR11_27752920	26.50247							
Obs_Htz38	12	Churra	OAR12_61804737	55.672814	s66802	55.842304	Australian Poll Merino	s66802	55.842304	OAR12_61974786	55.848797
		Milk Lacaune	OAR12_61874204	55.74348	OAR12_61974786	55.848797	Meat Lacaune	s66802	55.842304	OAR12_61974786	55.848797
		Comisana	s66802	55.842304			Australian Poll Merino				
Obs_Htz39	12	Churra	s30614	58.136309	OAR12_64628319	58.302665	Meat Lacaune	s30614	58.136309		
		East Frisian Brown	s26801	58.576013	OAR12_64944672	58.61688					
Obs_Htz40	13	Churra	OAR13_33752762	30.635254	OAR13_33955992	30.85995	Ojalada	OAR13_33752762	30.635254	OAR13_33896421	30.786161
		Milk Lacaune	OAR13_33896421	30.786161	OAR13_33955992	30.85995	Australian Poll Merino	OAR13_34032007	30.939655	OAR13_34176651	31.065051

Obs_Htz41	13	Milk Lacaune	s08503	39.88509							
		Comisana	OAR13_54016379	41.65332	s20479	41.694042					
Obs_Htz42	13	Chios	OAR13_60893851	56.060654	OAR13_68535533	63.227469	Sakiz	s31558	56.277903	s13874	63.323127
		Comisana	s31201	56.339588	s63708	63.78132	Finnsheep	OAR13_67067820	61.900545	s19346	62.210378
							Australian Poll Merino	s48133	62.276683	OAR13_67425309	62.371867
Obs_Htz43	15	Churra	OAR15_37160581	35.602806	OAR15_37380344	35.789479	Finnsheep	s65735	31.027264		
		Comisana	OAR15_37160581	35.602806	OAR15_37443121	35.84624					
Obs_Htz44	15	Chios	s02793	72.842543			Finnsheep	OAR15_77074005	71.384913	OAR15_77122668	71.432136
		Milk Lacaune	s28875	72.948461			Australian Poll Merino	s28875	72.948461	s52687	72.988365
							Meat Lacaune	s28875	72.948461		
							Ojalada	s28875	72.948461		
Obs_Htz45	16	Comisana	OAR16_51977009	48.03606							
		East Frisian Brown	OAR16_52089645	48.143409							
Obs_Htz46	17	Churra	OAR17_64627979	59.584736	s37924	60.05064	Meat Lacaune	OAR17_64889221	59.812538	s37924	60.05064
		Milk Lacaune	OAR17_64889221	59.812538	s37924	60.05064					
Obs_Htz47	20	Churra	OAR20_44626939	40.652715	s17843	40.959336	Meat Lacaune	OAR20_44626939	40.652715	s37017	40.920512
		Milk Lacaune	OAR20_44626939	40.652715	s37017	40.920512	Australian Poll Merino	s64274	41.01732		
Obs_Htz48	20	Churra	OAR20_54497778	49.766895	s35415	49.979989	Meat Lacaune	s61798	49.816123	s35415	49.979989
		Comisana	OAR20_54497778	49.766895	s35415	49.979989	Finnsheep	s35415	49.979989		
		Chios	s61798	49.816123	s35415	49.979989					
		Milk Lacaune	s61798	49.816123	s35415	49.979989					
Obs_Htz49	21	Chios	s52830	27.451792	OAR21_30678813	27.471775	Meat Lacaune	s75804	29.587175	s75804	29.587175
		Milk Lacaune	s41284	29.40318	s75804	29.587175	Australian Poll Merino	s38134	29.447881	s44540	29.507008
							Ojalada	s38134	29.447881	s44540	29.507008
Obs_Htz50	21	Churra	s72899	38.858968	s59728	38.91103	Australian Poll Merino	OAR21_46682445	40.738496	OAR21_46936641	40.874274
		Comisana	OAR21_46682445	40.738496							
		Milk Lacaune	OAR21_46682445	40.738496	OAR21_46936641	40.874274					
Obs_Htz51	22	Milk Lacaune	OAR22_23392099	19.587631	OAR22_23417873	19.610393	Meat Lacaune	OAR22_23392099	19.587631	OAR22_23446812	19.639123
		East Frisian Brown	OAR22_24747565	20.99124			Australian Poll Merino	OAR22_26275112	22.366773		
Obs_Htz52	22	Comisana	OAR22_36492428	31.879508	OAR22_40197865	35.61184	Ojalada	OAR22_36492428	31.879508	OAR22_36576021	31.963576
		Milk Lacaune	OAR22_36492428	31.879508	s73065	34.30344	Australian Poll Merino	OAR22_38946909	34.273821	OAR22_41042892	36.462598
							Meat Lacaune	OAR22_38946909	34.273821	s13462	38.550647
							Finnsheep	OAR22_42931135	38.322102	OAR22_43010940	38.403606
Obs_Htz53	24	Comisana	s08130	26.375802			Finnsheep	OAR24_28445680	26.035068	s63355	27.096216
		Milk Lacaune	s08130	26.375802	OAR24_29399883	26.984286	Ojalada	s08130	26.375802	s48918	26.58114
Obs_Htz54	25	Comisana	s12019	5.095615			Australian Poll Merino	s25195	6.235402	s67158	6.571187
		East Frisian Brown	s25195	6.235402	s21107	6.461437	Meat Lacaune	s44881	6.451855	s21107	6.461437
		Milk Lacaune	s03686	6.359903	s67158	6.571187					
Obs_Htz55	25	East Frisian Brown	OAR25_19926183	18.148995	OAR25_20142347	18.354065					
		Milk Lacaune	OAR25_20106030	18.316414							
