# Supplementary material for: Application of Selection Mapping to Identify Genomic Regions Associated with Dairy Production in Sheep
Source: PLoS One. 2014 May 1;9(5):e94623. doi: 10.1371/journal.pone.0094623 (PMC4006912; doi:10.1371/journal.pone.0094623)
Supplement: Table S4 — Candidate regions identified by the analysis based on observed heterozygosity (ObsHtz-CR), averaged in sliding windows of 9 SNPs (ObsHtz-9SNPW), that were exclusively detected in dairy breeds. (PDF) [file pone.0094623.s004.pdf]

Table S4 for

Application of selection mapping to identify genomic regions associated with dairy production in sheep

Authors: Beatriz Gutiérrez-Gil[1]*, Juan Jose Arranz[1], Ricardo Pong-Wong[2], Elsa García-Gámez[1], James Kijas[3], Pamela Wiener[2]

Table S4. Candidate regions identified by the analysis based on observed heterozygosity (ObsHtz-CR), averaged in sliding windows of 9 SNPs (ObsHtz-9SNPW), that were exclusively detected in dairy breeds. The interval of each region is referred (in bp) to the sheep genome reference sequence v2.0 (http://www.livestockgenomics.csiro.au/cgi-bin/gbrowse/oarv2.0/). The corresponding orthologous bovine genomic intervals are given based on the bovine genome reference sequence UMD 3.1 (http://www.ensembl.org/Bos_taurus/Info/Index). The positional candidate genes that map within the bovine candidate range and that are included as candidate genes for milk production and mastitis traits in the database provided by Ogorevc et al. [1] are indicated as functional candidate genes. The affected trait and reference in the SheepQTL and CattleQTL databases (at http://www.animalgenome.org/cgi-bin/QTLdb/index) for previously reported ovine and bovine QTL that map within the corresponding genomic regions and that influence milk production traits and some other functional traits related to dairy production are also indicated.

REFERENCES

[1] Ogorevc J, Kunej T, Razpet A, Dovc P (2009) Database of cattle candidate genes and genetic markers for milk production and mastitis. Anim Genet 40: 832-851.

ObsHtz-Candidate region only detected in dairy breeds	Sheep genome range (Mb) (Oar v2.0)	Bovine genome range (Mb) (UMD 3.1)	Functional candidate genes based on Ogorevc et al. [1]	QTL described in sheep (SheepQTLdb identifier[1])	QTL described in cattle in relation to milk production and functional dairy traits (CattleQTLdb identifier[2])	Nb. of positional candidates[3]
ObsHtz-CR3	OAR2: 20.132 – 20.274	BTA8: 94.200 – 94.210		Milk fat percentage (DYD) (13913)	Foot angle (3599), Stature (16279, 16280, 16281)	1
ObsHtz-CR7	OAR2: 180.309 – 183.404	BTA2: 67.936 – 70.143		Milk yield (14147), Milk lactose yield (13991)	Milk fat percentage (2650), Milk fat yield (2737), Milk protein yield (4494)	5
ObsHtz-CR8	OAR2: 199.479 – 199.814	BTA2: 86.439 – 86.694	*HSPD1*	Milk lactose yield (13991)	Palmitoleic acid content (5812), Milk protein yield (EBV) (6045, 9985), Milk fat percentage (daughter deviation) (9983), Milk protein percentage (daughter deviation) (9984)	6
ObsHtz-CR10	OAR2: 232.587 – 234.454	BTA2: 119.341 – 121.226			Somatic cell score (EBV) (6152)	28
ObsHtz-CR12	OAR3: 18.648 – 18.859	BTA11: 87.474 – 87.566			Milk fat yield (2669)	3
ObsHtz-CR25	OAR5: 40.407 – 41.107	BTA7: 43.939 – 44.939		Foot angle (14181)	Somatic cell score (2667)	29
ObsHtz-CR28	OAR7: 19.072 – 19.258	BTA10: 19.198 –19.317			Milk fat yield (10295). Milk protein yield (10297), Teat length (10296), Udder attachment (10294), Milk protein percentage (EBV) (10015)	2
ObsHtz-CR29	OAR7: 42.026 – 43.570	BTA10: 45.205 – 46.769		Milk protein percentage (13998)	Milk fat yield (daughter deviation) (10219), Milk yield (2554)	19

ObsHtz-CR31	OAR8: 78.041 – 78.759	BTA9: 92.429 –93.241			Milk fat yield QTL (1520), Clinical mastitis (10087), Milk protein percentage (2534), Milk protein yield (daughter deviation) (3752, 3755), Milk yield (daughter deviation) (3736, 3739)	6
ObsHtz-CR41	OAR13: 39.885 – 41.694	BTA13: 41.248 – 42.894			Milk protein percentage (2672), Rump angle (3429), Milk fat yield (2555)	17
ObsHtz-CR45	OAR16: 48.036 – 48.143	BTA20: 46.768 – 49.061			Milk fat percentage (EBV) (11329), Milk fat yield (EBV) (11328), Milk protein percentage (EBV) (11330), Udder attachment (10323), Udder depth (10322), Milk protein percentage (4805), Somatic cell score (13244)	0
ObsHtz-CR55	OAR25: 18.149 – 18.354	BTA28: 19.393 – 19.807		Milk Fat percentage (14010)	Milk protein percentage (EBV) (10017) Milk yield QTL (2691) Udder composite index (1663) Udder height (1661)	2

[1]Search QTL identifier number at http://www.animalgenome.org/cgi-bin/QTLdb/index to find complete details about the QTL reported in the sheep genomic region (SheepQTL database) and its corresponding orthologous bovine region (CattleQTL database) for the candidate region identified in this study.
