# Supplementary material for: Application of Selection Mapping to Identify Genomic Regions Associated with Dairy Production in Sheep
Source: PLoS One. 2014 May 1;9(5):e94623. doi: 10.1371/journal.pone.0094623 (PMC4006912; doi:10.1371/journal.pone.0094623)
Supplement: File S1 — Summary of the criteria for selection of breeds to be included in the study, including the results of a Principal Component Analysis (PCA) performed with the initial set of breeds considered. (PDF) [file pone.0094623.s005.pdf]

File S1 for

Application of selection mapping to identify genomic regions associated with dairy production in sheep

Authors: Beatriz Gutiérrez-Gil[1]*, Juan Jose Arranz[1], Ricardo Pong-Wong[2], Elsa García-Gámez[1], James Kijas[3], Pamela Wiener[2]

File S1. Summary of the criteria for selection of breeds to be included in the study, including the results of a Principal component analysis (PCA) performed with the initial set of breeds considered.

Selection of breeds for analysis

From the breeds analysed in the SheepHapMap project [1], a group of five European breeds was selected to be analysed as "dairy group" in the present study: Chios, Chura, Comisana, East Friesian Brown and Milk Lacaune (Table 1). This group included breeds showing different levels of dairy specialization (See Supporting Table 1 for additional breed information) with some highly-specialized dairy breeds such as East Friesian Brown and Milk Lacaune, and some others for which official dairy breeding improvement is more recent.

With the aim of providing an appropriate comparison set that could help identify the selection signals specifically related to dairy selection, we selected another group of non-dairy breeds to be included in the study. The initial selection of the non-dairy breeds was

based on the estimated divergence time between ovine breeds reported by Kijas et al. [1] from the extent of haplotype sharing that persists at increasing physical distances between SNP pairs (Supporting Information Figure S10 and Figure 3 of Kijas et al. [1]), choosing, for each dairy breed, the most closely related non-dairy breed to which it could be compared. Based on this, a group of six non-dairy breeds were selected, including both meat-specialized breeds and also breeds that have not been under specific selection pressure (i.e. traditional triple purpose breeds). For one of the dairy breeds, EastFriesianBrown, two potential comparison non-dairy breeds (Finnsheep and Scottish Texel) were initially investigated (Table 1). For the Milk Lacaune, in addition to a meat-specialized breed, the Australian Poll Merino, the Meat Lacaune variety was also considered for comparison. The Australian Poll Merino was also selected as comparative breed for the (dairy) Italian Comisana breed. The estimated divergence times between the initially selected pairs ranged from 80-160 generations (Milk Lacaune *vs* Meat Lacaune pair) and 480-560 (East Friesian Brown vs Finnsheep pair) [1].

The initial selection of non-dairy breeds was refined based on the results of a Principal Component Analysis (PCA) of allele sharing performed using *smartpca* implemented in Eigensoft [2] for the total list of breeds included in Table 1. Due to differences in the number of samples available for each breed, we selected 22 samples for each breed (the maximum number available for all selected breeds) to performed an N-balanced PCA analysis. This analysis helped to determine the clustering pattern within the initial selected dataset and, in conjunction with the haplotype similarities previously described [1], was used to choose related dairy and non-dairy breed pairs for subsequent genetic differentiation analysis.

This PCA analysis also included the Galway and Border Leicester breeds for comparison with the Scottish Texel breed in the pair-wise F_{ST} analysis of the myostatin gene region as a test case of a gene known to be under selection. According to Kijas et al. [1], these two breeds showed the closest phylogenetic relationship with Scottish Texel based on the extent of haplotype sharing (80-160 generations of divergence) and neither is known to carry the allele associated with muscle hypertrophy.

From this analysis, the proportion of variance explained by each component was obtained by dividing the eigenvalue corresponding to each component by the sum of all eigenvalues identified (for a total of 20 PC estimated).

Population structure analysis and selection of dairy and non-dairy breed pairs

The results of the PCA of allele sharing were plotted to show direct comparison of Principal Component 1 (PC1) against PC2 to PC5 (Supporting Figure S1). The two largest principal components (A) separated four differentiated clusters related to the geographical groups represented in the initial selected dataset: the mainland Mediterranean breeds (Lacaunes, Churra, Ojalada, Australian Poll Merino and Comisana), the island Mediterranean breeds (Chios, Sakiz and Cyprus Fat Tail), the mainland NorthEuropean breeds (Finnsheep and East Frisian Brown) and the island NorthEuropean breeds (Scottish Texel, Galway and Border Leicester). Based on this clustering, the Finnsheep was selected as the comparison breed for East Friesian Brown for subsequent analyses, rather than Scottish Texel.

The Australian Poll Merino showed a close relationship with all the other mainland Mediterranean breeds, and therefore was considered as the non-dairy breed to compare with

Milk Lacaune and Comisana, while the two Spanish breeds, Churra (dairy) - Ojalada (non-dairy), and the Mediterranean island breeds, Chios (dairy) - Sakiz (non-dairy), were considered as pairs. Also based on the PCA analysis, the Scottish Texel-Galway pair was selected for the pair-wise F_{ST} comparison for test-case control region selected in this study (myostatin), as these two breeds showed a closer relationship than that observed between the Scottish Texel and Border Leicester.

Based on this analysis, PC1 explained 18.93% of the genotypic variance, PC2 and PC3 explained 11.79% and 10.36% respectively, and PC4 and PC5 explained from 7.89% and 6.06% of the variance.

Figure S1: Clustering of animals based on principal component analysis of allele sharing for the initial selected breed dataset.

Individual animals from the initial selected breeds to include in the study are plotted for principal component (PC) 1 *vs* PC2 (A), for PC1 *vs* PC3 (B), for PC1 *vs* PC4 (C) and for te PCA1 *vs* PC5 (D). Individuals from different breeds are shown using different colored symbols as indicated in the legend.

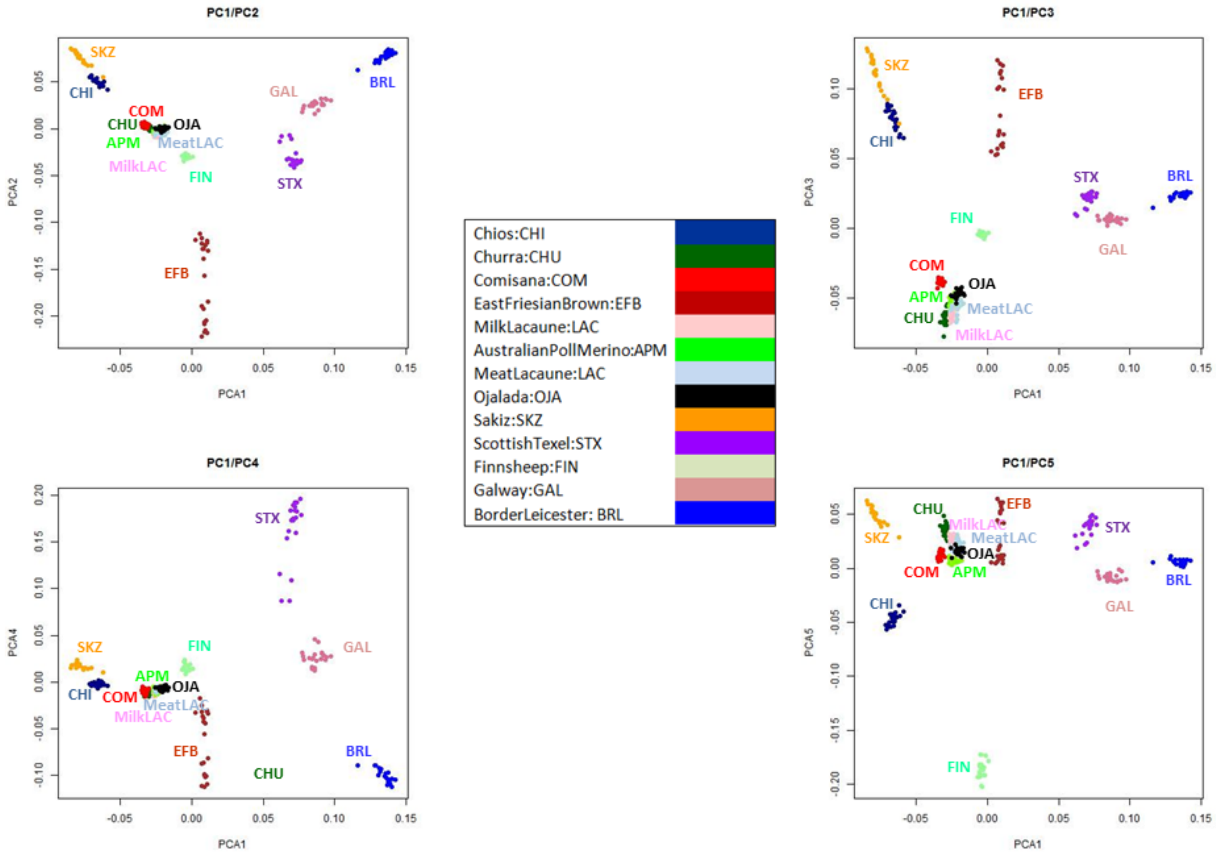

References

1. Kijas JW, Lenstra JA, Hayes B, Boitard S, Porto Neto LR, et al. (2012) Genome-wide analysis of the world's sheep breeds reveals high levels of historic mixture and strong recent selection. PLoS Biol 10: e1001258.
2. Patterson N, Price AL, Reich D. (2006) Population structure and eigenanalysis. PLoS Genet 2: e190.
