# Supplementary material for: Application of Selection Mapping to Identify Genomic Regions Associated with Dairy Production in Sheep
Source: PLoS One. 2014 May 1;9(5):e94623. doi: 10.1371/journal.pone.0094623 (PMC4006912; doi:10.1371/journal.pone.0094623)
Supplement: File S2 — Summary of the results of the analysis performed in this work in relation to the myostatin ( GDF-8 ) gene region. These results were evaluated to establish criteria for the analyses performed to detect dairy selection signatures in the dairy breeds. (PDF) [file pone.0094623.s006.pdf]

File S2 for

Application of selection mapping to identify genomic regions associated with dairy production in sheep

Authors: Beatriz Gutiérrez-Gil[1]*, Juan Jose Arranz[1], Ricardo Pong-Wong[2], Elsa García-Gámez[1], James Kijas[3], Pamela Wiener[2]

File S2: Summary of the results of the analysis performed in this work in relation to the myostatin (GDF-8) gene region. These results were evaluated to establish criteria for the analyses performed to detect dairy selection signatures in the dairy breeds analysed.

Methods: Test Case Analysis: Myostatin (*GDF-8*) Gene Region

The myostatin gene (*GDF*-8), variation at which is associated with muscle hypertrophy in the Belgian Texel breed [1] and other related sheep breeds [2], was considered as a test case to assess the ability of the different analyses to detect genomic regions that have been subject to selection pressure. This region was also used to evaluate the influence of parameters used for the analyses implemented in this study. Kijas et al. [3] showed that a selection signature in the *GDF*-8 gene region could be identified in three geographically distinct populations of Texel, including the Scottish Texel, which was considered as the reference breed for this test case assessment.

Based on the PCA analysis described in Supporting Information File 1, the Scottish Texel breed was compared to the Galway breed in the pair-wise F_{ST} analysis. Regions of low observed heterozygosity were also assessed for the Scottish Texel breed following the

methods described for the dairy and non-dairy breeds in the *Selection sweep mapping analysis methods* section of the main text of the manuscript. These analyses were implemented across the whole genome using sliding windows of 9-, 13- and 17- SNPs and the results obtained near the *GDF-8* gene (OAR2: 118.573 – 118.579 Mb) were evaluated to establish criteria for the analyses performed to detect dairy selection signatures. The results of the regression analysis for detection of regions with asymptotic heterozygosity patterns performed in the Scottish Texel breed, which was performed as described in the *Selection sweep mapping analysis methods* section of the main text of the manuscript, for the three tested bracket sizes (5, 10 and 20 Mbp) were also assessed for chromosome OAR2, in relation to the position of the *GDF-8* gene.

Results: Mapping accuracy of GDF-8 and setting of criteria for further analyses

Differentiation: For two of the window sizes (9- and 17-SNP) used to obtain the averaged F_{ST} for the Scottish Texel-Galway pair, the top result genome-wide was found in the OAR2 region carrying the *GDF-8* gene, whereas for the 13-SNP window the top position was found on OAR7 (33.45 Mb). The top location for the 9-SNP window size (at 115.28 Mb) was much closer to the actual position of *GDF-8* (118.57 Mb) than seen for the case of the 17-SNP window size (top position at 113.25 Mb). Based on these results, a 9-SNP window size was selected to calculate the average pair-wise F_{ST} values for the dairy *vs* non-dairy breed pairs. Furthermore, the distribution of identified positions near *GDF-8* was considered to determine the criteria to define a single selection signal. Among positions on OAR2 that were in the top 0.5 percent of the 9-SNP window F_{ST} (F_{ST}-9SNPW) values for the Scottish-Texel-Galway pair, 55 of 68 of them were found between 109.62 Mb and 122.62 Mb, with inter-marker distances less than or equal to 1.97 Mb (Supporting Figure S2a). The gaps flanking the

upstream and downstream positions to this interval of extreme genetic differentiation were 42.49 and 5.04 Mb respectively. Between the positions identified at 116.21 and 118.12 Mb, there was a gap of 1.91 Mb where no highly differentiated markers were detected. Based on these observations, the distance of 2 Mb was considered as the maximum interval between markers defining a single F_{ST}-based selection signal.

Reduced heterozygosity: In the Scottish Texel breed there was a region of decreased heterozygosity near the *GDF-8* gene detected with the three SNP window sizes tested. This region showed the lowest values of ObsHtz for 13 and 17-SNP window sizes, whereas the lowest value for the 9-SNP window size was found on OAR19, (0.047) followed by the signal around the myostatin region (0.056). For the three tested window sizes, decreased heterozygosity regions encompassed continuous markers positions on OAR2. The following regions included gaps up to 2.00 Mb: 108.88-119.51 Mb for Htz-9SNPW, 108.89-123.64 for Htz-13SNPW and 108.96-123.66 for Htz-13SNPW, all of which included *GDF-8* (118.573-118.579 Mb), such that the length of the low heterozogosity region increased with the window size. As the region of continuous low heterozygosity was smallest, the 9-SNP window size was selected to calculate the reduced diversity in the dairy and non-dairy breeds included in our study. The continuous region identified by Htz-9SNPW values near *GDF-8* (108.88-119.51 Mb) was flanked by gaps of 4.37 and 2.92 Mb long. Within that continuous region the maximum intermarker distance was 1.99 Mb upstream and 1.94 Mb downstream of the positions 111.39 and 113.77 Mb, respectively (Supporting Figure S2b). Hence, for this method up to a 2 Mb interval was again allowed within a region defined on the basis of the reduced heterozygosity values.

Asymptotic heterozygosity pattern: In the regression analysis for detection of regions with asymptotic heterozygosity patterns performed in the Scottish Texel breed, the *GDF-8* region had the highest –log(p) values across the whole genome for the two larger brackets, 10 and

20 Mbp, whereas for the 5 Mb-bracket, a region on OAR4 yielded the highest –log(p) value. The top position in the 10 Mb-bracket analysis (118.0598 Mb) was closest to the location of the *GDF-8* gene (Supporting Figure S2c). Based on this, results obtained with the 10 Mb-bracket for the dairy breeds were used for comparison with those obtained using genetic differentiation and observed heterozygosity. Although the gaps between identified positions within continuous regions were smaller than for the other methods, for consistency with the other two analyses, the same criterion was used (maximum distance between identified positions of 2 Mb) to determine candidate regions based on the asymptotic heterozygosity pattern.

Figure S2: Identification of the selection signature related to the *GDF-8* gene in OAR2 through the analysis of the Scottish Texel sheep breed following the three methodologies used in this work. I) Across-genome signals. a) Genome-wide distribution of F_{ST} values averaged in sliding windows of 9 SNPs (F_{ST}-9SNPW) obtained for the Scottish Texel-Galway breed pair. **b)** Genome-wide distribution of observed heterozygosity (ObsHtz) values averaged in sliding windows of 9 SNPs (ObsHtz-9SNPW) estimated for the Scottish Texel breed. **c)** Genome-wide distribution of –log(p) values resulting from the regression analysis for detection of regions with asymptotic heterozygosity patterns performed in the Scottish Texel breed and considering all markers within 10 Mb of this position (10 Mb-bracket size). **II) Plots of the 75-150 Mb region of OAR2 with details of the selection signature identified in the region of the *GDF-8* gene by the three considered methodologies: d)** ObsHtz-9SNPW; **e)** ObsHtz-9SNPW; **f)** Regression_10Mb-bracket size. The position of the *GDF-8* gene (OAR2: 118.573 – 118.579 Mb; v2.0) is indicated with a green arrow in the x-axis. The maker or position associated with the top or bottom value of the distribution is indicated in brown colour, whereas other markers are indicated in blue colour.

I)

a)

b)

c)

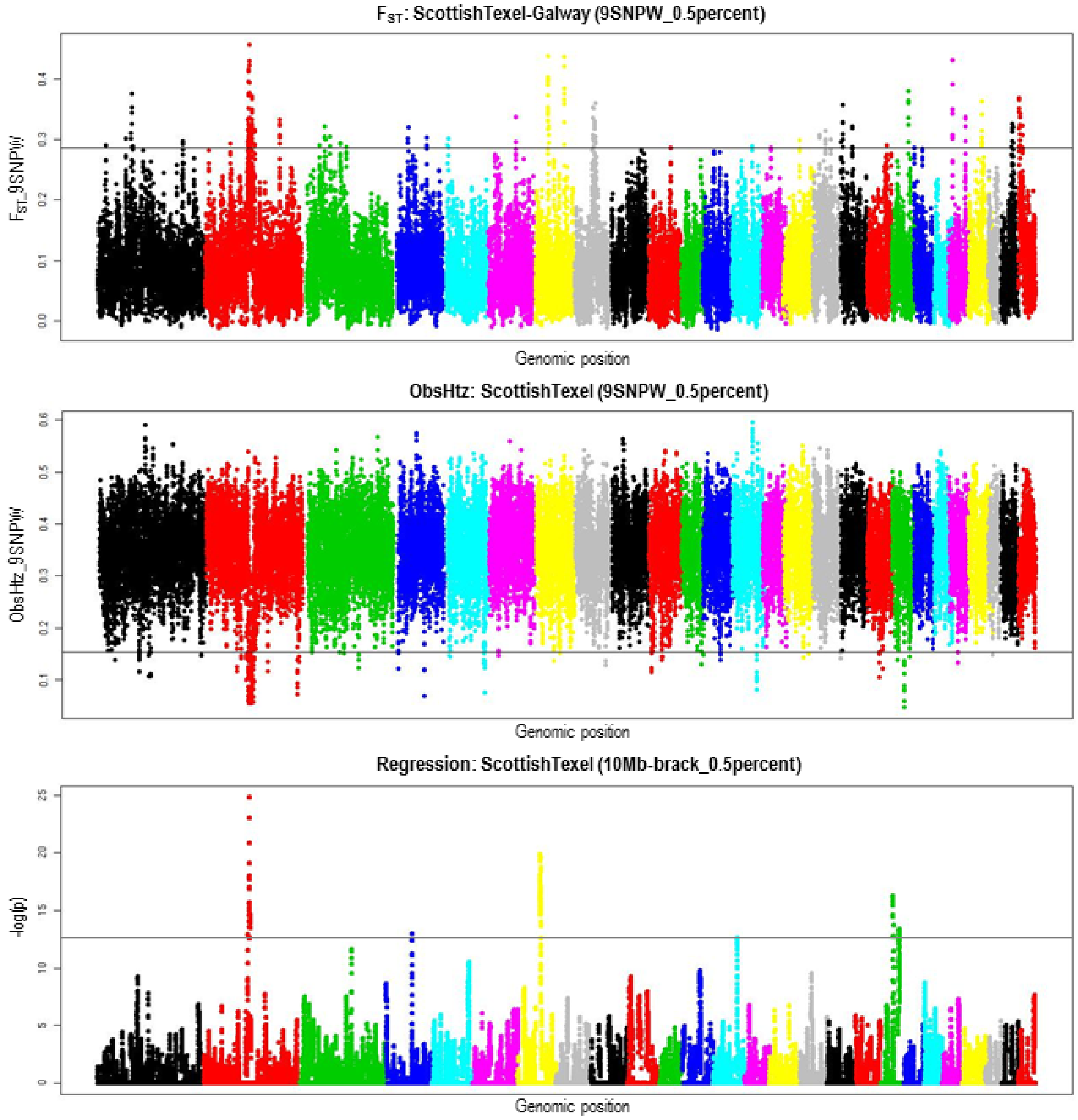

a) Genome-wide distribution of F_{ST} values for the ScottishTexel-Galway pair. The amount of genetic differentiation, measured as F_{ST}, was estimated within this breed pair, and averaged in sliding windows of 9 SNPs (F_{ST}-9SNPW) across the genome. The horizontal line indicates the top 0.5th percent threshold considered for the F_{ST}-distribution. The region showing the largest genetic differentiation was found in the OAR2 region carrying the *GDF-8* gene.

b) Genome-wide distribution of observed heterozygosity (ObsHtz) values estimated for the ScottishTexel breed, and averaged in sliding windows of 9 SNPs (ObsHtz-9SNPW) across the genome. The horizontal line indicates the bottom 0.5th percent threshold considered for the ObsHtz-distribution. The signal of decreased heterozygosity on OAR2 (interval 108.88-119.51 Mb) includes the *GDF-8* gene.

c) Genome-wide distribution of –log(p) values resulting from the regression analysis for detection of regions with asymptotic heterozygosity patterns performed in the Scottish Texel breed and considering all markers within 10 Mb of this position (10 Mb-bracket size). The –log(p) values were obtained for each test position which was moved every 50 Kb across each chromosome (-log(p) values were set to 0 where the asymptotic regression was not in the predicted direction, i.e. where $0<R<1$, $B<0$ was not met). The horizontal line indicates the top 0.5th percent threshold considered for the –log(p)-distribution. The most significant signal identified across the genome was found in the OAR2 region carrying the *GDF-8* gene.

II)

d)

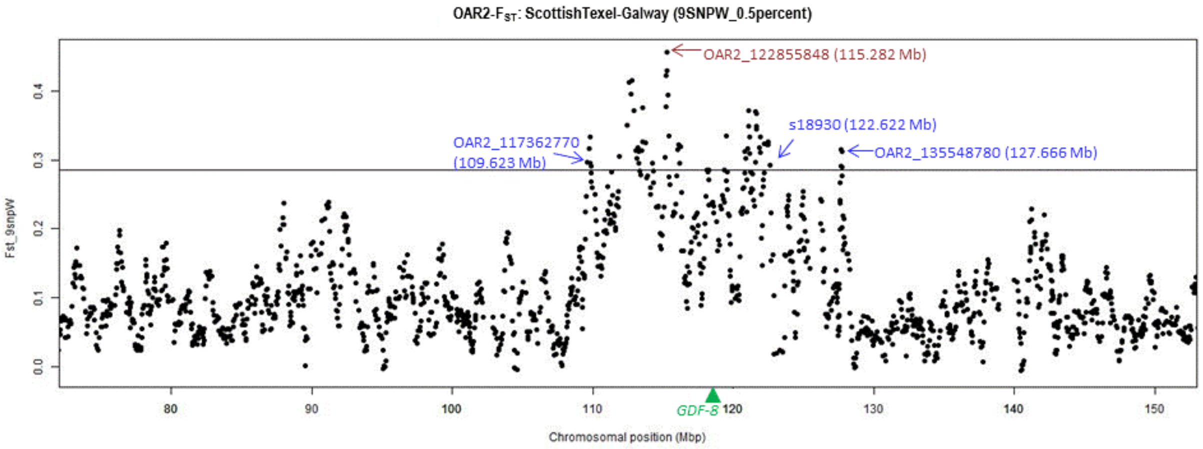

OAR2-F_{ST}: ScottishTexel-Galway (9SNPW_0.5percent)
OAR2_122855848 (115.282 Mb)
s18930 (122.622 Mb)
OAR2_117362770
(109.623 Mb)
OAR2_135548780 (127.666 Mb)
Fst_9snpW
GDF-8
Chromosomal position (Mbp)

e)

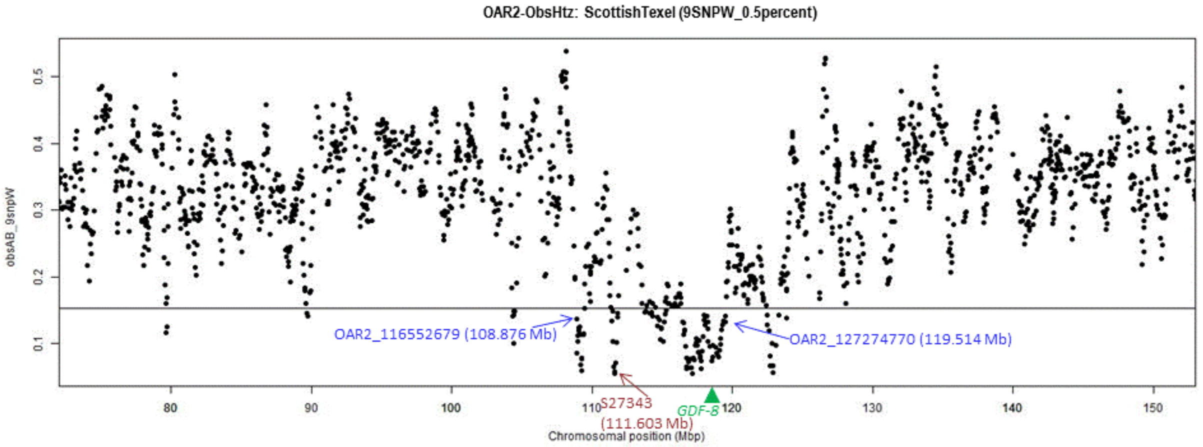

OAR2-ObsHtz: ScottishTexel (9SNPW_0.5percent)
obsAB_9snpW
OAR2_116552679 (108.876 Mb)
OAR2_127274770 (119.514 Mb)
S27343
(111.603 Mb)
GDF-8
Chromosomal position (Mbp)

f)

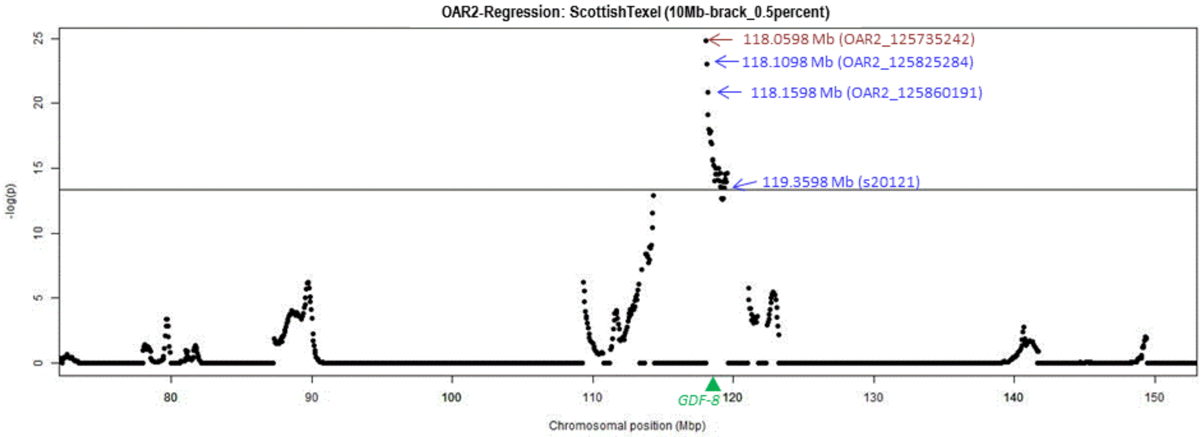

OAR2-Regression: ScottishTexel (10Mb-brack_0.5percent)
118.0598 Mb (OAR2_125735242)
118.1098 Mb (OAR2_125825284)
118.1598 Mb (OAR2_125860191)
119.3598 Mb (s20121)
-log(p)
GDF-8
Chromosomal position (Mbp)

REFERENCES

1. Clop A, Marcq F, Takeda H, Pirottin D, Tordoir X, et al. (2006) A mutation creating a potential illegitimate microRNA target site in the myostatin gene affects muscularity in sheep. Nat Genet 38: 813-818.
2. Bignell CW, Malau-Aduli AE, Nichols PD, McCulloch R, Kijas JW (2010) East Friesian sheep carry a Myostatin allele known to cause muscle hypertrophy in other breeds. Anim Genet 41: 445-446.
3. Kijas JW, Lenstra JA, Hayes B, Boitard S, Porto Neto LR, et al. (2012). Genome-wide analysis of the world's sheep breeds reveals high levels of historic mixture and strong recent selection. PLoS Biol 10: e1001258.
